# Investigation of the impact of dual inoculations of arbuscular mycorrhizal fungi and plant growth-promoting rhizobacteria on drought tolerance of maize grown in a compost-amended field under Mediterranean conditions

**DOI:** 10.3389/fmicb.2024.1432637

**Published:** 2024-10-09

**Authors:** Redouane Ouhaddou, Lahoucine Ech-chatir, Chayma Ikan, Fatima Ezzahra Soussani, Farid Errouh, Abderrahim Boutasknit, Julio Cesar Rodrigez, Salah Er-Raki, Robin Duponnois, Abdelilah Meddich

**Affiliations:** ^1^Center of Agrobiotechnology and Bioengineering, Research Unit Labelled CNRST (Centre AgroBiotech-URL-7 CNRST-05), Cadi Ayyad University, Marrakesh, Morocco; ^2^Plant Physiology and Biotechnology Team, Laboratory of Agro-Food, Biotechnologies and Valorization of Plant Bioresources (AGROBIOVAL), Department of Biology, Faculty of Science Semlalia, Cadi Ayyad University, Marrakesh, Morocco; ^3^Laboratory of Processes for Sustainable Energy and Environment (ProcEDE), Department of Applied Physics, Faculty of Science and Technology Guéliz, Cadi Ayyad University, Marrakesh, Morocco; ^4^Laboratory of Plant, Animal, and Agro-Industry Productions, Faculty of Science, University Ibn Tofail, Kenitra, Morocco; ^5^Department of Biology, Multidisciplinary Faculty of Nador, Mohammed First University – Oujda, Nador, Morocco; ^6^Departemento de Agricultura y Ganaderia, Universidad de Sonora, Hermosillo, Mexico; ^7^Center for Remote Sensing Applications (CRSA), Mohammed VI Polytechnic University (UM6P), Benguerir, Morocco; ^8^Laboratoire des Symbioses Tropicales & Méditerranéennes UMR 113 IRD/CIRAD/INRAe/SupAgro Montpellier/UM Campus International de Baillarguet TA A-82/J, Montpellier, France; ^9^African Sustainable Agriculture Research Institute (ASARI), University Mohammed VI Polytechnic (UM6P), Laayoune, Morocco

**Keywords:** climate change, water scarcity, soil poverty, symbiotic microbes, organic amendment, *Zea mays*, evapotranspiration, drip irrigation

## Abstract

In the current context of rapid climate change, water scarcity and soil poverty are becoming increasingly alarming, leading to growing losses of 30–50% of global agricultural production. It is imperative to find environmentally-friendly approaches for improving plant tolerance to drastic conditions, particularly in arid and semi-arid Mediterranean regions. Biostimulants based on symbiotic microbes are emerging as effective strategies for improving tolerance and agricultural productivity. This study aims to evaluate the effects of single and double inoculation of arbuscular mycorrhizal fungi (My) and plant growth-promoting bacteria (Ba) on the growth, physiological and biochemical traits of maize crop grown in compost (Co) amended soil under two irrigation regimes: well-watered (WW: 100% of crop evapotranspiration [ETc]) and drought-stressed (DS: 50% ETc) using drip irrigation system. Reducing irrigation to 50% reduced shoot dry weight (SDW), root dry weight (RDW), 1,000-grains weight (TGW) and grain yield (Y). However, Ba alone increased SDW by 63%, while CoMyBa improved RDW, TGW and Y by 197, 43 and 175%, respectively compared with the control under DS conditions. Dual inoculation boosted root colonization intensity, normalized difference vegetation index (NDVI), total chlorophyll and leaf area of maize seedlings in compost-amended soil, compared to the controls. The application of Ba significantly reduced hydrogen peroxide and malondialdehyde by 46%, in maize seedlings grown in compost-amended soil, compared to the controls under DS. Our results indicated that My and Ba significantly boost the ability of maize to tolerate drought by improving water supply and physiology and stimulating the accumulation of organic and inorganic osmolytes, as well as improving the properties of soils such as cation exchange capacity particularly amended by Co. The dual inoculations were the most effective and represent an environmentally-friendly and relatively inexpensive approach to optimizing agricultural production and soil restoration programs in Mediterranean regions.

## 1 Introduction

The world is currently facing a two-faceted challenge in the context of climate change: the increase in the world’s population and the concomitant rise in food demand. Mediterranean regions are most exposed to land degradation and desertification due to climatic challenges, making them more vulnerable. In addition, they have the lowest levels of organic matter and beneficial rhizospheric microorganisms, leading to soil sterility and imbalance in agricultural ecosystems. Furthermore, these regions are known for the scarcity and irregularity of precipitation, which goes hand in hand with increasing drought and high levels of evapotranspiration (Ylla et al., 2010; del Pozo et al., 2019; Su et al., 2020; Naveed et al., 2021). According to several scientific reports, drought is limiting agricultural productivity worldwide (Baslam and Goicoechea, 2012; Gupta et al., 2020; Soares et al., 2023). Its seriousness lies in the fact that it will affect more than 20% of arable land while increasing the need for food from 59 to 98% by 2050 (Bouwman et al., 2017; FAO, 2019). Moreover, water scarcity has serious and negative repercussions on the productivity and yield of a variety of crops (Shirinbayan et al., 2019). The worst is when it comes to crops of economic interest and which represent the staple foods for humans and animals, notably cereals (maize, wheat, barley, and quinoa) and horticulture crops: (tomatoes and lettuce) (Al-Naggar et al., 2017; Anwaar et al., 2020; Fracasso et al., 2020; Meddich et al., 2021; Balbaa et al., 2022; Ouhaddou et al., 2023a). The negative impact of this constraint lies in the fact that it affects the most critical phenological stages of plants, such as flowering, fruiting and grain filling (del Pozo et al., 2019). These losses caused by drought are essentially due to the malfunctioning of physiological, biochemical and nutritional traits, in particular: (1) the photosynthetic apparatus, (2) the antioxidant system, and (3) the absorption of water and mineral elements (Grant, 2012). Chlorophyll destruction results in low photosynthetic activity in the reaction centers of photosystem II (PS II), which reduces biomass and crop yield (Valizadeh et al., 2014; Maia Júnior et al., 2020). In addition, dry soil causes oxidative stress to plants due to the accumulation of reactive oxygen species (ROS), which weakens the plant’s enzymatic defense system (Kar, 2011; Qureshi et al., 2018). Responding to the food demands of a growing population through the application of chemical fertilizers has led to improved yields per unit area in the agricultural sector. Nevertheless, the intensive use of inorganic fertilizers in world agriculture to ensure global food security has caused numerous health problems and irreparable environmental pollution (Bai et al., 2020; Dhankhar and Kumar, 2023). In order to mitigate the effects of drought without damaging the health of the soil and humans, biological approaches based on natural biostimulants are recommended.

Rhizosphere micro-organisms are used for this purpose because of their ability to promote plant growth under conditions of drought stress (Sorty et al., 2018; Vidal et al., 2022). Arbuscular mycorrhizal fungi (AMF) are known for their capacity to supply the plant with its needs via the network of hyphae (Wipf et al., 2019; Pepe et al., 2020). The establishment of symbiosis with the plant’s roots positively modifies the plant’s mineral and water status, especially in dry, phosphorus-poor soils (Cera et al., 2021; Tshibangu Kazadi et al., 2022). They increase plant tolerance to abiotic stresses by strengthening the enzymatic antioxidant system and improving stomatal conductance, leading to greater biomass (Wu and Zou, 2017). Plant growth-promoting bacteria (PGPR) also play an important role in improving plant growth and tolerance to abiotic stress (Khoshru et al., 2020). Bacteria of the genus *Bacillus* are the most dominant rhizospheric microorganisms in regions suffering from water shortage (Etesami et al., 2023). This beneficial effect is due to PGPR characteristics such as the synthesis of growth hormones (auxin), chelation of iron by siderophores, solubilization of potassium and phosphorus, synthesis of the enzyme 1-aminocyclopropane-1-carboxylic acid (ACC) deaminase, and resistance to drought stress (Wahid et al., 2022; Etesami et al., 2023). The tolerance of plants to drought stress via the application of AMF and/or PGPR is also explained by osmotic adjustment by accumulating organic substances called osmolytes such as proline (Yooyongwech and Phaukinsang, 2013; Chun et al., 2018). Recent research has highlighted the potential for synergistic interactions between AMF and PGPR when used in double inoculation (Aini et al., 2019). The co-inoculation of AMF and PGPR into crops has been shown to increase biomass, boost yields and improve tolerance to abiotic stresses (Diagne et al., 2020). In addition, the mechanisms behind these synergistic effects of AMF and PGPR on crops were elucidated. PGPR can enhance AMF spore germination and hyphal growth, leading to more efficient mycorrhizal colonization (Gopal et al., 2012; Sagar et al., 2021). This mutual enhancement can lead to a more robust root system, able to better absorb nutrients and water, ultimately promoting plant growth and resistance (Wahid et al., 2022). In addition to microorganisms, enriching the soil with organic soil improvers such as compost improves its structure and composition in terms of mineral elements, especially phosphorus and nitrogen, as well as organic matter (Boutasknit et al., 2020). Compost has been shown to improve plant growth under drought-stress conditions because of its water retention capacity (Liu et al., 2012). The introduction of compost into agricultural ecosystems is an alternative to chemical fertilizers (Pascual et al., 2004). Compost increases plant resistance in water-limited conditions by triggering various mechanisms such as antioxidant enzymes, the accumulation of osmolytes (proline, sugars, glycine betaine, etc.) and improved gas exchange (Fiasconaro et al., 2019; Anli et al., 2020; Ahanger et al., 2021; Akensous et al., 2023). It has been investigated that the combined application of these biostimulants improves plant tolerance to abiotic stresses such as drought. The synergistic effect between AMF and PGPR bacteria has already been validated by several scientific reports in terms of the bioavailability of nutrients assimilated by the plant, the encouragement of symbiosis, improved fruit quality and crop yields under controlled conditions and in the field (Aini et al., 2019; Raklami et al., 2019; Tahiri et al., 2022). Compost presents an organic matrix and a source of nutrients that promotes the rapid growth of PGPR (Cozzolino et al., 2016). These bacteria, in their role of releasing mineral elements from their immobile form (solubilization of phosphorus and potassium), facilitate uptake by plants (Grobelak et al., 2015). Improved soil structure (aeration, porosity…, etc.) and water supply to roots via AMF hyphal extensions, as well as increased root architecture through the auxinic action of PGPR, considerably enhance plant growth and resilience to abiotic stresses (Zhang et al., 2018).

Morocco’s economy relies heavily on agriculture and its food security is based on cereals, which occupy 55% of the agricultural area (Schilling et al., 2012). Maize is a crucial cereal for this security contributing about 12.5% to gross gomestic groduct (GPD) (Belmahi et al., 2023). Maize cultivation in Morocco plays an important role in the economic sector due to its consumption by humans and animals (Achli et al., 2022). Maize (*Zea mays* L. Saccharata) is a variety known for its high sugar content due to spontaneous mutation (Revilla et al., 2021). It is rich in bioactive molecules like vitamins (Liu et al., 2017; Xiang et al., 2019). It is one of the most sensitive cereal crops to water shortage (Revilla et al., 2021). Maize’s water requirements vary between 500 and 700 mm, for which the standard crop coefficients during three main stages are: Kc_ini_ = 0.3, Kc_mid_ = 1.15, and Kc_end_ = 1.05 (Allen et al., 1998). Due to its shallow root system, maize is considered to be sensitive to drought stress (Moussa et al., 2008; Song et al., 2019). It has been reported that the yield of maize was reduced to 37% when irrigation was limited to 30% (Singh et al., 2022). Drought stress at grain filling can reduce maize yield by 3% per day of water shortage, indicating the sensitivity of maize to delayed irrigation (Lauer, 2003). Recently, Moroccan researchers validated the performance of these microorganisms on an organic matrix in terms of the tolerance of different crops under controlled and field conditions (Boutasknit et al., 2021; Anli et al., 2022; Benaffari et al., 2022; Ouhaddou et al., 2023a; Soussani et al., 2023). In contrast, the application of these biostimulants in compost-amended soils under irrigation control based on crop evapotranspiration (ETc) has not yet been exploited. The application of this technology in open field conditions on water-stressed maize is not yet well exploited. Consequently, the aim of this study is to examine the performance of biostimulants on the morphological, physiological and biochemical adaptation as well as tolerance and yield of maize crop under two water levels based on ETc. We hypothesized that: (i) dual inoculation of AMF and PGPR applied to compost-amended soil can improve growth, physiology, biochemistry, yield and drought stress tolerance in the field; (ii) biostimulants can offer an alternative to the excessive use of chemical fertilizers (N-P-K) under water stress conditions; and (iii) biostimulants can positively modify soil physicochemical properties such as cation exchange capacity, available phosphorus and glomalin content compared with N-P-K application under both water regimes.

## 2 Materials and methods

### 2.1 Experimental site

**Figure 1:**
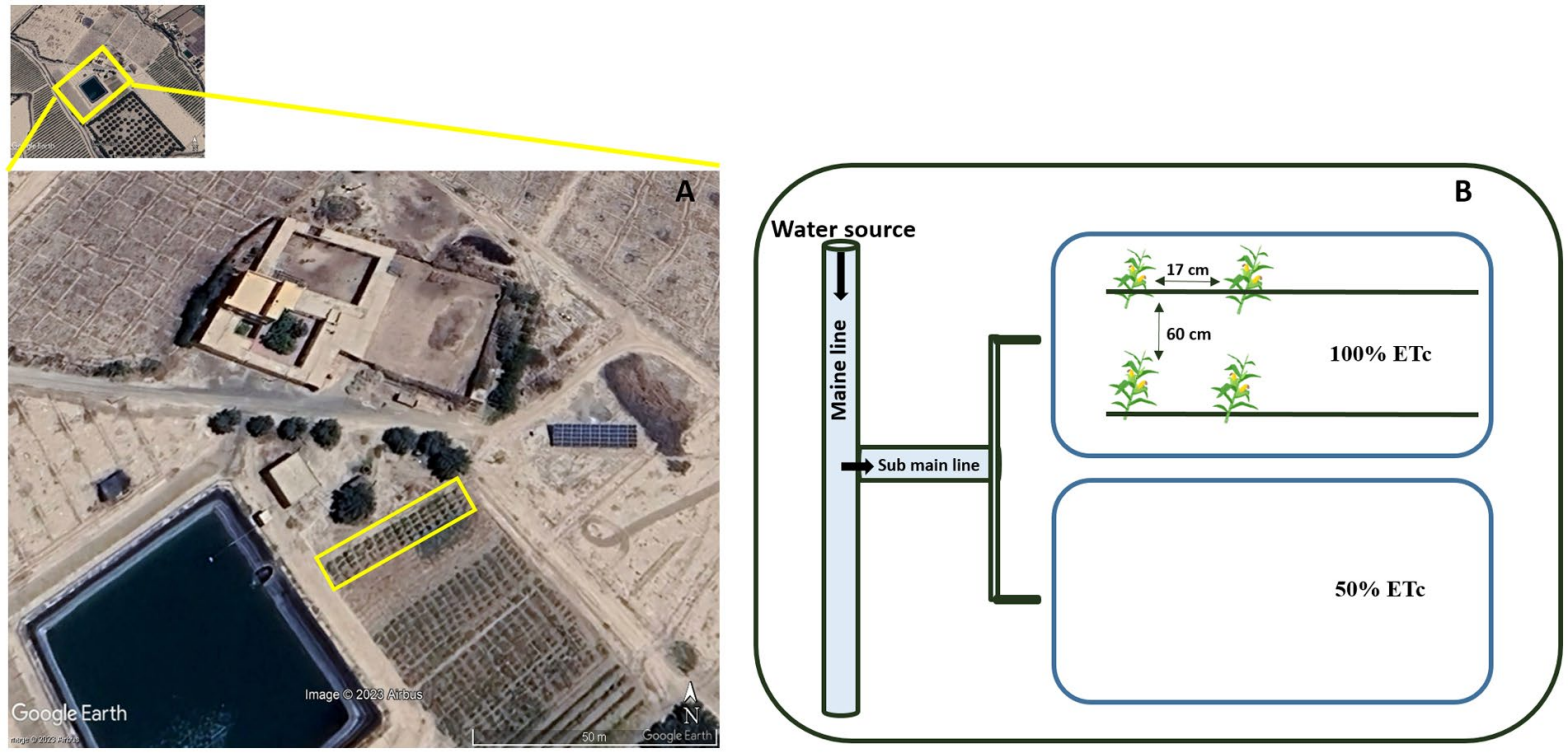
**(A)** Experimental site map and **(B)** design.

The experiment was carried out in 2023 at the agricultural field in the commune of Loudaya 35 km from Marrakech, Morocco, at the following geographical coordinates 31°34′46.2″N, 8°16′13.1″W (Figure 1A). The climate of this region is semi-arid with maximum and minimum average annual temperatures of 30.1°C and 15.3°C, respectively as well as 98.29 mm of total annual precipitation. Soil samples were taken from a depth of 0–30 cm before the cultivation. The physicochemical characteristics of the soil revealed a pH of 8.36, electrical conductivity (EC) of 0.38 mS/cm, cation exchange capacity (CEC) of 4.48 meq/100 g, permittivity of 3.6, organic matter of 1.78%, P-Olsen of 98.2 mg/kg, N of 0.76%, bulk density of 1.39 g/cm^3^ and a sandy clay loam texture (loam 24%, clay 32, and 44%). It should be noted that the maize was grown in accordance with good agricultural practices and that the experimental field was never previously treated with conventional chemical fertilizers.

### 2.2 Experimental design and treatments

A two-block design (Figure 1B) was used in this study in the form of plots designated for 18 treatments with three replications. Each block consisted of one water regime and two rows of plots. Each experimental plot had a surface area of 1.2 m^2^ (1.50 m long and 0.80 m wide) and a spacing of 0.60 m between plots. Maize (*Zea mays* L. Saccharata) seeds were sown on 03 March, 2023 and a plant spacing of 0.17 m with two rows/plot spaced 0.60 m apart. Using reference evapotranspiration (ET0) and the maize crop coefficient (Kc_ini_ = 0.3, Kc_mid_ = 1.15, and Kc_end_ = 1.05) in accordance with Allen et al. (1998), we imposed two irrigation regimes based on maize crop water requirement (ETc) according to the following formula:

ETc=ETo∗Kc

- ETc: Crop evapotranspiration (mm/day), which is the quantity of water lost by a specific crop through both evaporation and transpiration.
- ET₀: Reference evapotranspiration (mm/day), calculated for a standard, well-watered grass surface. It is independent of the crop.
- Kc: Crop coefficient (without unit), which adjusts the ET₀ to reflect the crop’s specific characteristics, such as its growth stage and plant cover.

A drip irrigation system was used. The first block of maize plants was watered well at 100% ETc (WW) using 8 L/h drippers and the second corresponded to drought stress (DS) with 50% ETc maintained by the 4 L/h drippers. In parallel, the treatments were applied individually or in combination as follows:

- Ctr−: negative control, untreated plants.
- Ctr+: positive control, plants treated only by N-P-K, 165-60-60 kg ha^−1^, respectively.
- Ba: plants treated by the bacterial consortium of plant growth promoting rhizobacteria.
- My: plants treated by arbuscular mycorrhizal fungi consortium.
- Co: plants treated by compost.
- MyBa: plants treated by arbuscular mycorrhizal fungi consortium and the bacterial consortium of plant growth promoting rhizobacteria.
- BaCo: plants treated by the bacterial consortium of plant growth promoting rhizobacteria and compost.
- CoMy: plants treated by compost and arbuscular mycorrhizal fungi consortium.
- CoMyBa: plants treated by compost, arbuscular mycorrhizal fungi consortium and the bacterial consortium of plant growth promoting rhizobacteria.

Treatments were distributed randomly within each block. At the 4–5 leaf stage of the maize plants, DS was applied and the soil water content was maintained according to the desired water regime. Weeds were controlled manually throughout the growing season.

### 2.3 Biostimulants characteristics and application

Two microbial consortia isolated from the rhizosphere of the Tafilalet palm grove (31°41′20.3″N 4°10′44.7″W), a semi-arid region located 500 km southeast of Marrakech, Morocco, were used in this study. The first is based on bacteria that promote plant growth: *Bacillus subtilis* and *Bacillus* sp. (Ba). These strains were isolated from collected soil, mixed with a sterile 0.09% NaCl solution and incubated for 30 min. Serial dilutions were made and aliquots of dilutions (10^−5^ and 10^−6^) were put on the surface of the National Botanical Research Institute’s phosphate growth medium devoid of yeast extract (NBRIY) medium. Plates were incubated for 48 h at 30°C. Colonies were purified by replating on fresh TSA medium to obtain single colonies. The characterization of the both bacteria is described in Table 1. These bacteria have been molecularly identified based on amplification of the 16S ribosomal RNA (rRNA) gene of *B*. sp. and *B. subtilis* which refer to sequence ID: AM981260.1 and MT457467.1 respectively, which aligns and matched with the GenBank database using the NCBI. After purification, each strain was grown in Tryptic Soy Broth (TSB) liquid medium under agitation for 48 h at 30°C to an optical density of approximately 1 at 600 nm, corresponding to 10^9^ CFU/mL. Plant inoculation was carried out by adding 10 mL of the bacterial suspension formed from the two above-mentioned strains in equal volumes to the root zone (Agbodjato et al., 2015). A booster of the same volume was applied after 15 days to ensure infection of the newly formed roots. The second is based on arbuscular mycorrhizal fungi (AMF) composed of 15 species, 60% of which are from the *Glomus* sp. and 20% from the *Acaulospora* sp. (Benaffari et al., 2022) (Supplementary Table 1). The identification of these species was based on the morphological appearance of the spore in terms of color, shape, size, germination shield, bulb, spore suspensor and membrane separation (Giovannetti and Gianinazzi-Pearson, 1994; Yano-Melo and Maia, 2010). Spores were identified on the basis of criteria proposed by Schenck et al. (1982) and descriptions supplied by the International Culture Collection of Vesicular Arbuscular Mycorrhizal^1^ following the classification of Redecker et al. (2013). 5 g of AMF were used as inoculum (hyphae, vesicles, roots and substrate containing spores) for the maize plants (Abdullahi et al., 2018). The product of composting local green waste was used as an organic amendment (Meddich et al., 2016). The quantity of compost applied was 4 Mg ha^−1^ at a rate of 480 g/plot (Tahiri et al., 2022). Compost was applied by incorporation into the soil at a depth of 5–10 cm before sowing. The physicochemical characteristics of the compost based on dry matter are: available phosphorus: 700 ppm, nitrogen: 1.5%, potassium: 0.5%, organic carbon: 20%.

**Table 1:** Characteristics of bacterial strains used.

Characteristics	*Bacillus* sp.	*Bacillus subtilis*
Phosphate solubilization index	2.39 ± 0.63	2.06 ± 0.30
Phosphate solubilization (mg/L)	82.11 ± 1.84	68.48 ± 0.77
Potassium solubilization index	2.11 ± 0.25	2.62 ± 0.29
Exopolysaccharide production (mg of CR/OD600)	89.33 ± 9.63	317.44 ± 20.45
IAA production (μg/mL)	444.55 ± 4.11	347.18 ± 3.07
Resistance to polyethylene glycol 6000	+	−

−, absent; +, presence; CR, Congo Red; IAA, indole-3-acetic acid.

### 2.4 Determination of mycorrhization and effect on plant growth

After harvest, the roots of the maize plants were cleaned, treated and stained with trypan blue to visualize the mycorrhizal structures according to the methodology developed by Phillips and Hayman (1970). After microscopic examination (OPTIKA microscopes, Italy), the mycorrhization frequency (MF) and mycorrhization intensity (MI) were measured as described by Trouvelot et al. (1986).

The agronomic parameters of maize plants, including shoot dry weight (SDW), root dry weight (RDW), shoot height (SH), root length (RL), stem diameter (SD), cob fresh weight (CFW), cob length (CL), cob diameter (CD), grains lines number per cob (GLC), grains number per cob (GNC), thousand grains weight (TGW), and yield per hectare (Y) were measured.

### 2.5 Determination of plant physiological activities

#### 2.5.1 Stomatal conductance, chlorophyll fluorescence, and chlorophyll pigment determination

Stomatal conductance (g_s_) was measured between 09:00 and 11:00 using a porometer (CI-340, Handheld Photosynthesis System, Washington, DC, USA) on well-developed leaves along the leaf blade according to the procedure described by Harley et al. (1992).

Chlorophyll fluorescence was measured using a portable fluorometer: OPTI-SCIENCE, OS30p (Hudson, NY, USA). The operation consisted of creating darkness on the leaves for 30 min using clips. A flash of light in 1 s was then used to record initial fluorescence (F_0_), maximum fluorescence (F_m_) and quantum yield (F_m_ − F_0_)/F_m_ = F_v_/F_m_, where F_v_ is the variable fluorescence (Baker, 2008). Photosynthetic pigments were quantified using the method of Arnon (1949).

#### 2.5.2 Vegetation indices

Leaf area (LA) was measured following the method developed by Radford (1967).

$$LA=k\;\left(L*W\right)$$

Where, LA = leaf area (cm^2^); K = constant (0.75); L = leaf length (cm) and W = maximum leaf width (cm).

The Trimble GreenSeeker (HCS100, Trimble Inc., Sumnyvale, CA, USA) is a portable spectrometer (optical sensor) used to measure the normalized difference vegetation index (NDVI) values. The spectrometer is supposed to be placed 60 cm above the maize plants. Its field of view has an ecliptical shape that widens as the sensor is held higher. The sensor emits brief pulses of red and infrared light and then measures the amount of each type of light reflected by the plant. The sensor continues to sample the scanned area as long as the trigger is held down. When the trigger is released, the sensor displays the measured value as an NDVI reading (ranging from 0.00 to 0.99) on its display screen for 10 s (Trimble, 2022).

### 2.6 Biochemical analysis

#### 2.6.1 Antioxidant activity

Polyphenol oxidase (PPO) activity was measured according to the method of Hori et al. (1997). 0.1 mL of enzyme extract was mixed with 2 mL of catechol (10 mM) in phosphate buffer (pH 7). PPO activity was expressed as enzyme unit mg^−1^ protein. One unit of PPO activity was defined as the amount of enzyme causing an increase in absorbance of 0.001 min^−1^ at 420 nm.

The activity of catalase (CAT) was measured as a decrease in absorbance at 240 nm for 3 min following the decomposition of H_2_O_2_ (Aebi, 1984). The solution contained 0.1 M potassium phosphate buffer (pH 7.0), 0.1 mM EDTA, 20 mM H_2_O_2_ and 100 μL of enzyme extract in a 2 mL volume. Enzyme activity is expressed in EU/mg MF/min, then in EU/mg protein/min. One EU (enzyme unit) is considered to correspond to the variation of 0.001 OD units.

#### 2.6.2 Proline and total soluble sugars

The total soluble sugar (TSS) content was determined according to the method of Dubois et al. (1956). Extraction was carried out by grinding 0.1 g in ethanol (80%) (*v/v*). After centrifugation, 1 mL of concentrated sulfuric acid and 0.2 mL of phenol were added to 0.2 mL of the recovered supernatant. The mixture was read at 485 nm using a spectrophotometer.

The proline content was determined using the method of Carillo et al. (2008). 0.1 g of fresh material was ground in 4 mL of 40% ethanol (*v/v*). The extract was placed at 4°C overnight. Next, 1 mL of a mixture of 60% acetic acid, 1% ninhydrin and 20% ethanol was added to 0.5 mL of the ethanolic extract obtained. The reaction mixture was placed at 90°C for 20 min. The optical density (OD) was then read at 520 nm.

#### 2.6.3 Malondialdehyde and hydrogen peroxide

The quantification of malondialdehyde (MDA) was determined using the method developed by Dhindsa and Matowe (1981). 1 mL of acetone (90%) and 1 mL of trichloroacetic acid (TCA) (10%) were used to homogenize 0.05 g of fresh material. After centrifugation, 0.25 mL of the supernatant was added to 0.5 mL of 0.1% phosphoric acid and 0.5 mL of 0.6% thiobarbituric acid (TBA). All stoppered tubes were incubated at 100°C for 30 min and then placed in an ice bath to stop the reaction. Next, 0.75 mL of 1-butanol was added. The coloration formed was measured at 532 and 600 nm.

Hydrogen peroxide (H_2_O_2_) levels in the leaves were determined by grinding 0.1 g in 2 mL of 10% (w/v) TCA. After centrifugation, 0.5 mL of the supernatant was mixed with 0.5 mL potassium phosphate buffer (10 mM, pH 7) and 1 mL potassium iodide (1 M). Readings were taken at 390 nm (Velikova et al., 2000).

### 2.7 Post-harvest plant physicochemical analysis and soil glomalin

Samples taken from the root zone (10–40 cm) of maize plants were subjected to the following analyses: Nitrogen (N) was assessed using the Kjeldahl method, 0.5 g of soil, 5 mL of concentrated sulfuric acid and 0.5 g of Kjeldahl catalyst were placed into a matron. Boiled until the samples turned white and then cooled under the host. The mineralizate was recovered in 100 mL distilled water for distillation. A control was made under the same conditions. Distillation was carried out using a Kjeldahl distiller (KJA-9840 Model, China). Titration was done with sulfuric acid (0.02 N) using a few drops of Tachero indicator. Available phosphorus (AP) was measured following the methodology of Olsen and Sommers (1982). 1 g of soil was added to 20 mL of 0.5 M sodium bicarbonate (NaHCO_3_), and stirred for 1 h then filtered. Then, 1 mL filtrate was mixed with 4 mL distilled water and 5 mL AB reagent, placed in a water bath for 10 min, and allowed to cool, then spectrophotometer readings at 820 nm were taken. Total organic carbon (TOC) and total organic matter (TOM) were assessed by the procedure of Aubert (1978), and electrical conductivity (conductivity meter, HI-9033, Hanna Instruments, Padova, Italy) and pH (pH meter, HI 9025) were also measured. The cation exchange capacity (CEC) was calculated using the method of Zuquette (1987).

The total glomalin-related soil protein (T-GRSP) was quantified according to the method of Cornejo et al. (2008). A 50 mM sodium citrate buffer (pH 8.0) containing 4 mL was used to extract the T-GRSP from 1 g of dry soil. The extract was centrifuged at 10,000×*g* for 1 h and autoclaved for 1 h at 121°C. The Bradford assay was used to assess the T-GRSP content (Bradford, 1976).

### 2.8 Statistical analysis

Statistical analyses were performed using CoSTAT version 6.3 software (developed by, Cohort software, Berkeley, CA, USA). Statistical studies based on the Honest Significant Difference (HSD) test, were performed by Tukey’s test with a significant value of 5%. At the *p* < 0.05 level, smaller values indicate significant differences between treatments (five repetitions). Correlation (Pearson’s correlation coefficient), principal component analysis (PCA), parallel coordinates plots (PCP) and hierarchical ascending classification (HAC), were performed using R Studio software (version 2023.9.1.494) in order to group all information concerning the physiological and biochemical phenotypic traits of the maize plants as well as physicochemical properties of soil treated or not with biostimulants under 100 and 50% ETc.

## 3 Results

### 3.1 Effect of biostimulants on root mycorrhization and plant growth

**Figure 2:**
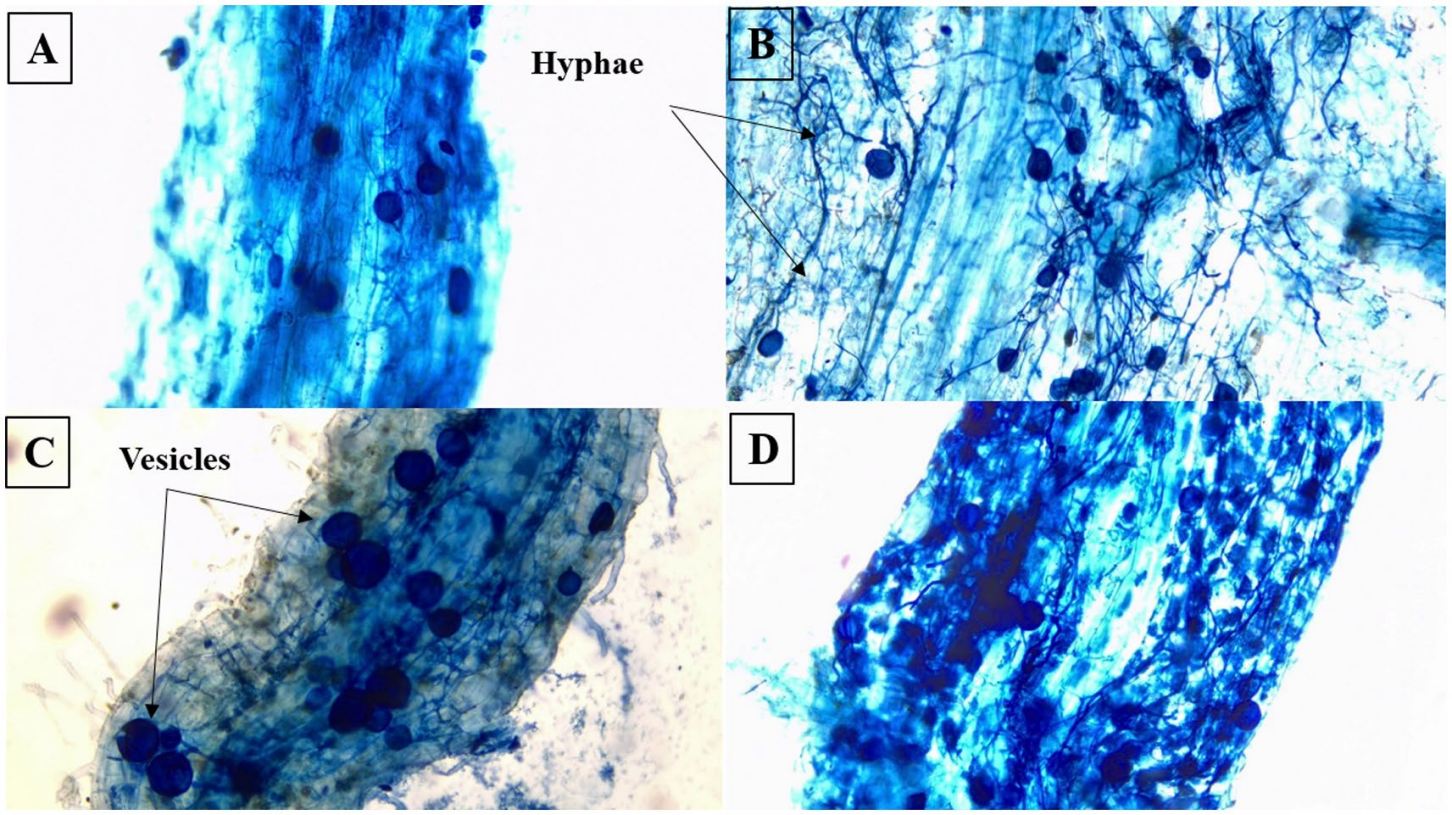
Microscopic observation (×100) of mycorrhizal structures in **(A)** untreated plants, **(B)** plants treated by arbuscular mycorrhizal fungi, **(C)** plants treated by arbuscular mycorrhizal fungi and plant growth promoting rhizobacteria and **(D)** plants treated by compost and arbuscular mycorrhizal fungi and plant growth promoting rhizobacteria.

Mycorrhization intensity (MI) of the roots increased under drought stress (DS) conditions than under normal irrigation conditions (Table 2; Figures 2A–D). Plants double-inoculated and grown in compost-amended soil (CoMyBa) showed a MI of around 50% whatever the irrigation imposed in comparison with all the treatments (Figure 2D). In fact, inoculation of plant (compost-amended or not) with Ba increased root MI compared with non-inoculated Ctr− (Figures 2A–C). In contrast, the application of N-P-K (Ctr+) to the soil significantly reduced MI by 16% compared with Ctr−. As for mycorrhization frequency (MF), it was always 100% whatever the water regime applied, except for Ctr− where MF was 96% under WW conditions.

**Table 2:** Effect of two irrigation levels and biostimulants on phenotypic and mycorrhization traits of maize plants.

Treatments	Shoot dry weight (g)		Root dry weight (g)		Shoot height (cm)		Root length (cm)		Stem diameter (mm)		Mycorrhization intensity (%)		Mycorrhization frequency (%)	
	WW	DS	WW	DS	WW	DS	WW	DS	WW	DS	WW	DS	WW	DS
Ctr−	103.6 ± 23.9 ^bc^	91.5 ± 9.3 ^bc^	11.8 ± 1.8 ^de^	6.8 ± 1.0 ^e^	161.6 ± 10.2 ^b-e^	125.4 ± 9.1 ^e^	24.8 ± 2.1 ^c^	22.4 ± 1.5 ^c^	22.2 ± 0.6 ^de^	20.5 ± 0.8 ^e^	18.4 ± 0.4 ^cd^	30.7 ± 3.0 ^a-d^	96.6 ± 1.9 ^b^	100 ± 0.0 ^a^
Ctr+	183.2 ± 19.2 ^ab^	116.9 ± 21.8 ^a-c^	27.9 ± 5.3 ^a-c^	16.6 ± 1.4 ^c-e^	199.6 ± 6.1 ^ab^	172.4 ± 5.4 ^b-d^	39.6 ± 5.8 ^a^	26.8 ± 2.0 ^bc^	23.2 ± 0.6 ^c-e^	23.5 ± 0.6 ^b-e^	23.3 ± 5.9 ^b-d^	16.8 ± 2.7 ^d^	100 ± 0.0 ^a^	100 ± 0.0 ^a^
Ba	209.3 ± 16.9 ^a^	149.4 ± 16.5 ^a-c^	34.8 ± 1.2 ^a^	17.5 ± 3.1 ^c-e^	230.0 ± 9.2 ^a^	161.8 ± 7.8 ^b-e^	37.4 ± 1.6 ^ab^	27.5 ± 1.8 ^a-c^	27.0 ± 0.4 ^a-c^	21.7 ± 1.0 ^de^	27.1 ± 3.6 ^b-d^	33.0 ± 3.8 ^a-d^	100 ± 0.0 ^a^	100 ± 0.0 ^a^
My	144.5 ± 9.3 ^a-c^	91.2 ± 7.9 ^bc^	21.2 ± 2.5 ^a-d^	12.7 ± 1.2 ^de^	177.4 ± 11.9 ^b-d^	153.2 ± 7.1 ^c-e^	33.4 ± 1.0 ^a-c^	26.4 ± 2.4 ^bc^	23.0 ± 0.7 ^c-e^	21.7 ± 0.7 ^de^	22.1 ± 0.9 ^cd^	39.5 ± 8.1 ^a-d^	100 ± 0.0 ^a^	100 ± 0.0 ^a^
Co	182.6 ± 31.1 ^a-c^	124.5 ± 9.6 ^a-c^	28.5 ± 5.5 ^a-c^	18.5 ± 2.5 ^c-e^	190.2 ± 4.8 ^a-c^	148.8 ± 6.3 ^de^	29.8 ± 3.4 ^a-c^	24.6 ± 2.4 ^c^	23.3 ± 1.0 ^c-e^	21.4 ± 0.7 ^de^	30.2 ± 0.6 ^a-d^	30.2 ± 0.1 ^a-d^	100 ± 0.0 ^a^	100 ± 0.0 ^a^
MyBa	133.2 ± 30.5 ^a-c^	120.0 ± 10.2 ^a-c^	18.5 ± 1.3 ^c-e^	12.5 ± 1.6 ^de^	166.8 ± 3.9 ^b-d^	143.0 ± 12.8 ^de^	26.0 ± 1.3 ^bc^	25.6 ± 1.6 ^bc^	26.7 ± 1.0 ^a-c^	24.6 ± 0.4 ^a-d^	30.6 ± 1.2 ^a-d^	42.4 ± 9.4 ^a-c^	100 ± 0.0 ^a^	100 ± 0.0 ^a^
BaCo	126.1 ± 12.4 ^a-c^	102.9 ± 5.6 ^bc^	13.8 ± 2.6 ^de^	10.6 ± 1.2 ^de^	166.0 ± 5.6 ^b-d^	164.2 ± 4.8 ^b-e^	26.6 ± 2.1 ^bc^	24.6 ± 1.2 ^c^	27.4 ± 1.0 ^ab^	26.2 ± 0.6 ^a-c^	17.1 ± 1.6 ^d^	34.6 ± 2.6 ^a-d^	100 ± 0.0 ^a^	100 ± 0.0 ^a^
CoMy	119.0 ± 2.9 ^a-c^	80.7 ± 4.9 ^c^	19.0 ± 4.3 ^b-e^	9.5 ± 2.5 ^de^	164.8 ± 8.4 ^b-e^	158.2 ± 5.1 ^c-e^	29.8 ± 2.5 ^a-c^	23.0 ± 1.3 ^c^	24.3 ± 0.6 ^a-e^	21.0 ± 0.8 ^de^	34.6 ± 6.1 ^a-d^	26.5 ± 7.2 ^b-d^	100 ± 0.0 ^a^	100 ± 0.0 ^a^
CoMyBa	210.8 ± 44.9 ^a^	145.5 ± 10.9 ^a-c^	32.9 ± 1.5 ^ab^	20.2 ± 1.2 ^b-e^	221.6 ± 3.5 ^a^	161.2 ± 9.3 ^b-e^	31.4 ± 2.1 ^a-c^	27.8 ± 1.7 ^a-c^	27.8 ± 0.5 ^a^	23.2 ± 0.8 ^c-e^	52.5 ± 5.5 ^a^	46.8 ± 2.3 ^ab^	100 ± 0.0 ^a^	100 ± 0.0 ^a^

WW, well-watered; DS, drought-stressed; Ctr−, untreated plants; Ctr+, plants treated by NPK; Ba, plant growth promoting rhizobacteria; My, arbuscular mycorrhizal fungi; Co, compost; MyBa, arbuscular mycorrhizal fungi and plant growth promoting rhizobacteria; BaCo, plant growth promoting rhizobacteria and compost; CoMy, compost and arbuscular mycorrhizal fungi; CoMyBa, compost and arbuscular mycorrhizal fungi and plant growth promoting rhizobacteria.

Values are means ± SE for five biological replicates. The values of each column labeled with different letters indicate significant differences assessed by Tukey’s test (*p* < 0.05). Data were taken at the reproductive/milk stage and after harvesting.

**Figure 3:**
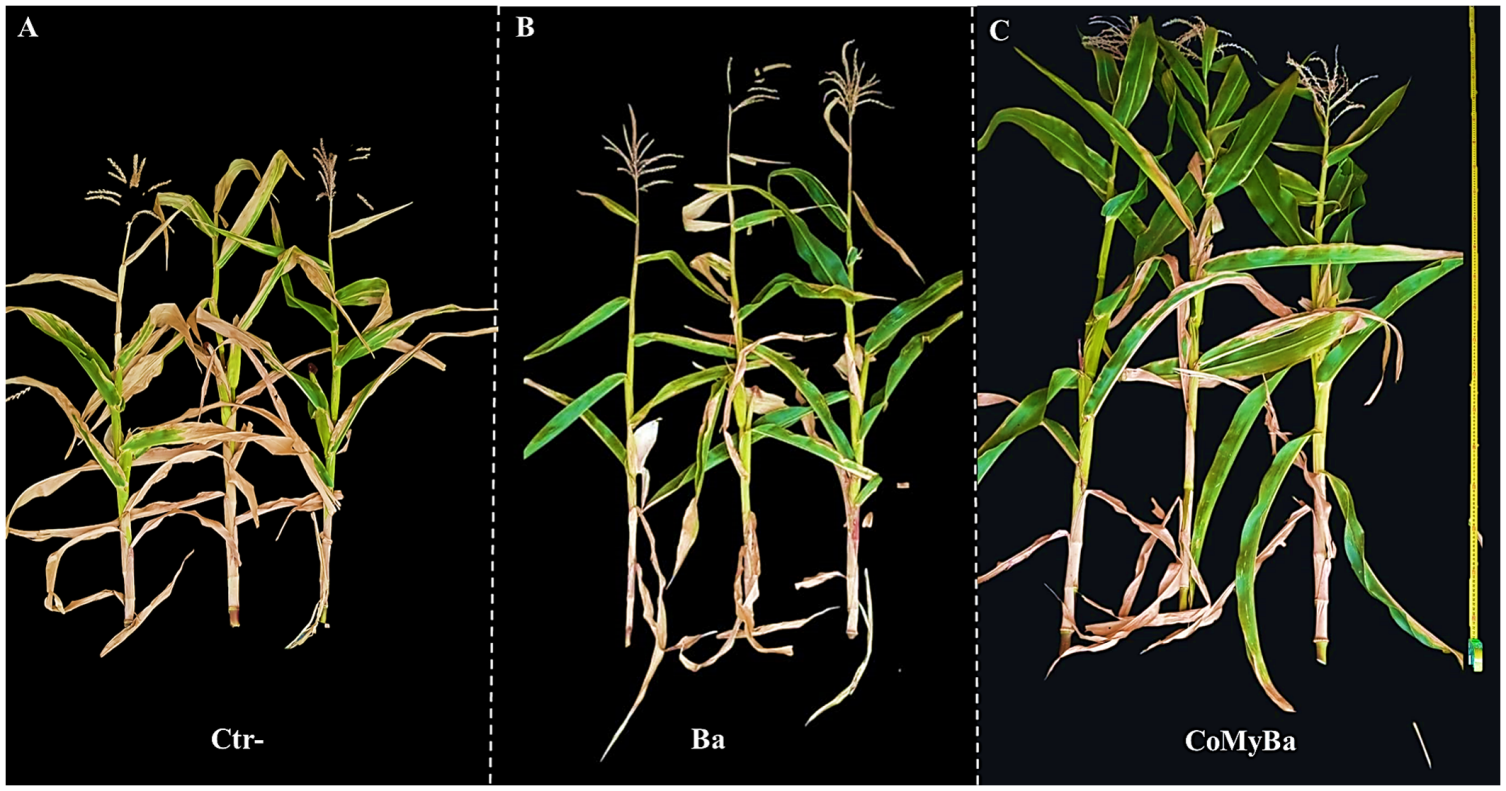
Illustration of the effect of drought stress (50% ETc) and biostimulants on morphological traits of maize plants. **(A)** Ctr−, untreated plants; **(B)** Ba, plants treated by plant growth promoting rhizobacteria; **(C)** CoMyBa, plants treated by compost and arbuscular mycorrhizal fungi and plant growth promoting rhizobacteria.

In terms of phenotypic traits, the water regime corresponding to 50% of ETc affected a range of growth parameters. However, soil enrichment with biostimulants stimulated maize growth, particularly shoot dry weight (SDW), root dry weight (RDW), shoot height (SH), root length (RL), stem diameter (SD), fresh cob weight (CFW), cob length (CL), cob diameter (CD), number of cobs formed (CN), and number of grain lines per cob (GLC) compared to the of 100% ETc (Tables 2, 3; Figures 3A–C). Whereas Ctr+ (plants treated only by N-P-K) achieved only 76% improvement compared with Ctr− (untreated plants) under the same conditions. Under 100% ETc, treatment of maize plants inoculated with Ba separately and in combination with My in the presence of compost (CoMyBa) significantly improved SDW by 102.02 and 103.46%, respectively compared to the non-compost amended and non-inoculated plants (Ctr−). While Ctr+ achieved 76% improvement compared to Ctr− under the same conditions. However, under 50% ETc, this parameter was highest in plants treated with Ba, with a 63% improvement for all treatments. In fact, Ba inoculation without compost improved RDW highly (194%) than double inoculation of maize plants with Ba and My in the presence of compost (178%) compared to Ctr− under ETc conditions of 100%. Furthermore, under 50% ETc, plants that were doubly inoculated and grown in compost-amended soil (CoMyBa) recorded higher root biomass than plants that were neither inoculated nor amended. Treatment of the plants with N-P-K (Ctr+) significantly (*p* < 0.05) increased SH under DS compared with all treatments, including Ctr−. The effect of biostimulants on RL under DS was exceptional in plants inoculated with microbes in the presence of compost compared to other treatments not treated with compost. The SD was significantly (*p* < 0.05) improved by BaCo and MyBa treatments, 28 and 20%, respectively under 50% ETc compared with their respective controls and the double combination in amended soil.

**Table 3:** Effect of two irrigation levels and biostimulants on yield traits of maize plants.

Treatments	Cob length (cm)		Cob diameter (mm)		Cob fresh weight (g)		Grain line/cob		Grains number/cob		Thousand grains weight (g)		Yield (Mg ha^−1^)	
	WW	DS	WW	DS	WW	DS	WW	DS	WW	DS	WW	DS	WW	DS
Ctr−	15.6 ± 1.1 ^b-d^	14.6 ± 0.1 ^d^	38.2 ± 1.6 ^a^	37.8 ± 1.3 ^a^	132.5 ± 18.1 ^cd^	116.3 ± 9.0 ^d^	11.8 ± 0.4 ^a^	11.6 ± 0.7 ^a^	352.4 ± 26.8 ^ab^	295.3 ± 56.1 ^b^	292.7 ± 5.1 ^ab^	229.5 ± 15.8 ^b^	6.6 ± 0.1 ^ab^	3.8 ± 1.1 ^b^
Ctr+	16.7 ± 0.8 ^a-d^	16.7 ± 0.5 ^a-d^	42.9 ± 1.4 ^a^	41.5 ± 1.6 ^a^	173.7 ± 2.5 ^a-d^	180.1 ± 9.3 ^a-c^	12.6 ± 0.1 ^a^	12.6 ± 0.8 ^a^	407.4 ± 23.7 ^ab^	371.8 ± 10.2 ^ab^	347.5 ± 5.6 ^ab^	291.5 ± 28.1 ^ab^	10.5 ± 1.1 ^ab^	8.4 ± 1.5 ^ab^
Ba	18.3 ± 0.3 ^a^	17.5 ± 0.6 ^a-c^	40.0 ± 0.4 ^a^	39.3 ± 1.4 ^a^	170.5 ± 10.6 ^a-d^	151.2 ± 9.4 ^a-d^	13.0 ± 0.2 ^a^	12.2 ± 0.5 ^a^	402.4 ± 34.5 ^ab^	367.0 ± 16.8 ^ab^	339.5 ± 2.5 ^ab^	313.3 ± 28.8 ^ab^	11.2 ± 1.9 ^ab^	9.1 ± 1.5 ^ab^
My	18.3 ± 0.1 ^a^	16.0 ± 0.2 ^a-d^	43.7 ± 0.5 ^a^	38.5 ± 2.8 ^a^	195.0 ± 9.0 ^ab^	134.2 ± 24.5 ^b-d^	13.0 ± 0.5 ^a^	12.3 ± 0.4 ^a^	439.4 ± 38.1 ^ab^	390.5 ± 31.2 ^ab^	342.2 ± 28.0 ^ab^	310.6 ± 57.3 ^ab^	13.8 ± 3.0 ^a^	7.6 ± 0.8 ^ab^
Co	17.8 ± 0.1 ^a-c^	15.2 ± 0.5 ^cd^	40.4 ± 0.9 ^a^	40.8 ± 1.8 ^a^	178.2 ± 6.8 ^a-d^	146.4 ± 7.2 ^a-d^	12.5 ± 0.2 ^a^	13.0 ± 0.5 ^a^	383.7 ± 13.4 ^ab^	353.8 ± 30.6 ^ab^	300.6 ± 15.3 ^ab^	306.7 ± 15.6 ^ab^	9.0 ± 1.2 ^ab^	6.8 ± 1.4 ^ab^
MyBa	17.0 ± 0.1 ^a-d^	16.7 ± 0.2 ^a-d^	41.1 ± 0.8 ^a^	41.6 ± 0.35 ^a^	168.5 ± 1.5 ^a-d^	175.2 ± 3.5 ^a-d^	12.6 ± 0.1 ^a^	12.4 ± 0.3 ^a^	442.0 ± 5.6 ^ab^	386.6 ± 22.0 ^ab^	281.0 ± 21.9 ^ab^	256.1 ± 34.4 ^b^	11.5 ± 0.5 ^ab^	7.4 ± 0.3 ^ab^
BaCo	16.8 ± 0.2 ^a-d^	16.4 ± 0.5 ^a-d^	41.7 ± 0.2 ^a^	39.1 ± 1.32 ^a^	164.8 ± 3.0 ^a-d^	136.9 ± 8.4 ^b-d^	13.0 ± 0.2 ^a^	12.6 ± 0.1 ^a^	412.5 ± 31.0 ^ab^	343.3 ± 32.9 ^ab^	308.5 ± 38.0 ^ab^	306.4 ± 21.7 ^ab^	9.6 ± 1.0 ^ab^	7.8 ± 2.5 ^ab^
CoMy	16.6 ± 0.5 ^a-d^	15.5 ± 0.6 ^b-d^	41.1 ± 0.9 ^a^	38.1 ± 1.4 ^a^	176.2 ± 25.2 ^a-d^	131.1 ± 10.3 ^cd^	12.3 ± 0.6 ^a^	12.8 ± 0.1 ^a^	366.1 ± 53.5 ^ab^	358.1 ± 28.3 ^ab^	317.7 ± 9.4 ^ab^	269.3 ± 15.3 ^ab^	7.8 ± 0.7 ^ab^	6.2 ± 0.5 ^ab^
CoMyBa	18.1 ± 0.0 ^ab^	16.9 ± 0.1 ^a-d^	44.1 ± 0.7 ^a^	41.0 ± 1.9 ^a^	199.2 ± 11.0 ^a^	170.0 ± 2.8 ^a-d^	13.3 ± 0.2 ^a^	12.6 ± 0.4 ^a^	467.2 ± 16.5 ^a^	452.4 ± 37.8 ^ab^	385.4 ± 6.2 ^a^	328.8 ± 12.5 ^ab^	13.2 ± 1.7 ^a^	10.6 ± 1.9 ^ab^

WW, well-watered; DS, drought-stressed; Ctr−, untreated plants; Ctr+, plants treated by NPK; Ba, plant growth promoting rhizobacteria; My, arbuscular mycorrhizal fungi; Co, compost; MyBa, arbuscular mycorrhizal fungi and plant growth promoting rhizobacteria; BaCo, plant growth promoting rhizobacteria and compost; CoMy, compost and arbuscular mycorrhizal fungi; CoMyBa, compost and arbuscular mycorrhizal fungi and plant growth promoting rhizobacteria.

Values are means ± SE for five biological replicates. The values of each column labeled with different letters indicate significant differences assessed by Tukey’s test (*p* < 0.05). Data were taken at the reproductive/milk stage and after harvesting.

It should also be noted that the biostimulants had a positive effect on maize cob parameters (Table 3). Indeed, an improvement of CFW was highly noted under WW when plants were cultivated in soil-amended Co combined with microbes. In contrast, this parameter improved by MyBa without Co compared with their controls. Compared with their unstressed controls, My and/or Ba without and with Co, were the most distinctive treatments (*p* < 0.05) for the improvement in CL. However, in the DS conditions, the values of this parameter were higher in plants inoculated with Ba without Co than in its presence with microbes compared with Ctr−. It should be noted that inoculation with MyBa had a beneficial effect on CD compared with Ctr− under DS conditions. Under the same conditions and compared with Ctr−, GLC was improved in plants grown in soil amended with Co alone or in combination with microbial consortia. Double microbial inoculation in the presence of Co positively increased GN whatever the water regime imposed compared to maize plants grown in soil free of Co and Ctr−.

The yield of plants exposed to DS was negatively affected compared with plants interacting with biostimulants applied alone or in combination (Table 3). The TGW was highest in plots where plants received the dual inoculation and grown in Co amended soil (CoMyBa) than treated with N-P-K, My and Ba, i.e., 31, 18.70, 16.88, and 16%, respectively under 100% ETc compared to the Ctr−. On the other hand, under DS this parameter was positively influenced by dual inoculation and grown in compost-amended soil by 43.27% compared to the Ctr−. At the end of the maize plant cycle, maximum yield per hectare (Mg ha^−1^) was obtained in plots treated with My without Co (13.83 ± 3.07, 106%) compared with those treated by MyBa in amended soil (13.27 ± 1.72, 97.83%) and Ctr−. Conversely, under water-limiting conditions, values were highest in plots treated with the double combination in amended soil (CoMyBa) than in a single application of Ba without Co: 10.65 ± 1.97, 175% and 9.11 ± 1.53, 136%, respectively.

### 3.2 Physiological analysis

#### 3.2.1 Stomatal conductance and chlorophyll fluorescence

**Figure 4:**
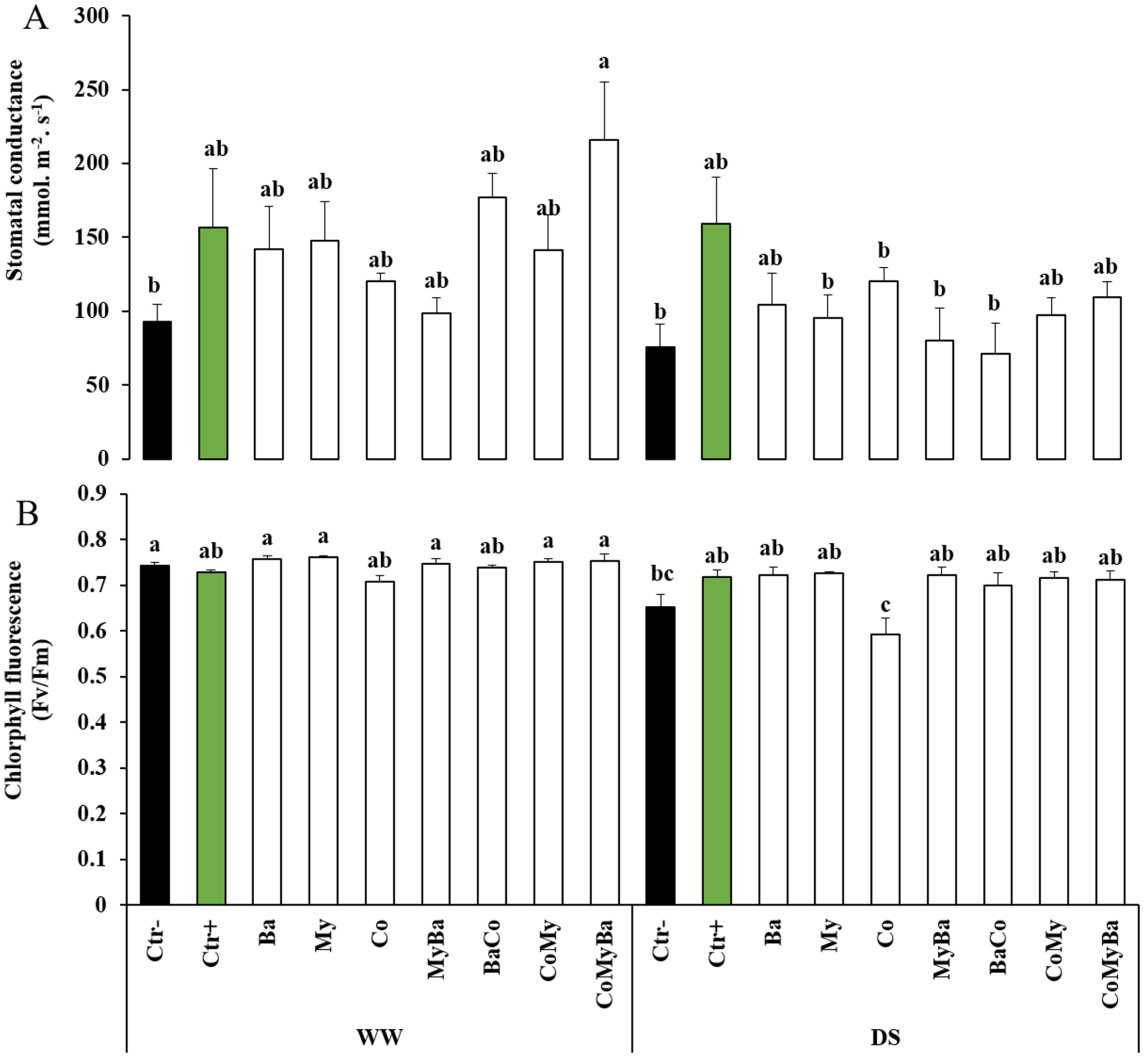
Effect of two irrigation levels and biostimulants on **(A)** stomatal conductance and **(B)** chlorophyll fluorescence of maize plants. WW, well-watered; DS, drought-stressed; Ctr−, untreated plants; Ctr+, plants treated by NPK; Ba, plant growth promoting rhizobacteria; My, arbuscular mycorrhizal fungi; Co, compost; MyBa, arbuscular mycorrhizal fungi and plant growth promoting rhizobacteria; BaCo, plant growth promoting rhizobacteria and compost; CoMy, compost and arbuscular mycorrhizal fungi; CoMyBa, compost and arbuscular mycorrhizal fungi and plant growth promoting rhizobacteria. Means (±SE; 5 biological replicates) followed by the same letters are not significantly different at *p* < 0.05 (Tukey’s HSD). Data were taken at the reproductive/milk stage.

Figure 4 shows that the physiological traits of maize plants were influenced by their exposure to DS and by the introduction of biostimulants applied alone or in interaction. In fact, stomatal conductance (g_s_) showed an increase in plants treated with Ctr+ compared to all treatments under 50% ETc or 100% ETc. The combined action of My and Ba in soil added by Co significantly improved g_s_ by 133.28% than other treatments compared to the Ctr− WW conditions. The same combination was effective in improving g_s_ as well as Ba without Co when the soil was desiccated (50% ETc) compared to the Ctr− (Figure 4A). As for chlorophyll fluorescence (Fv/Fm), the My and Ba treatments were distinctive compared with the other treatments by 11.34 and 10.83%, respectively under 100% ETc.

#### 3.2.2 Photosynthetic pigments and carotenoids

**Figure 5:**
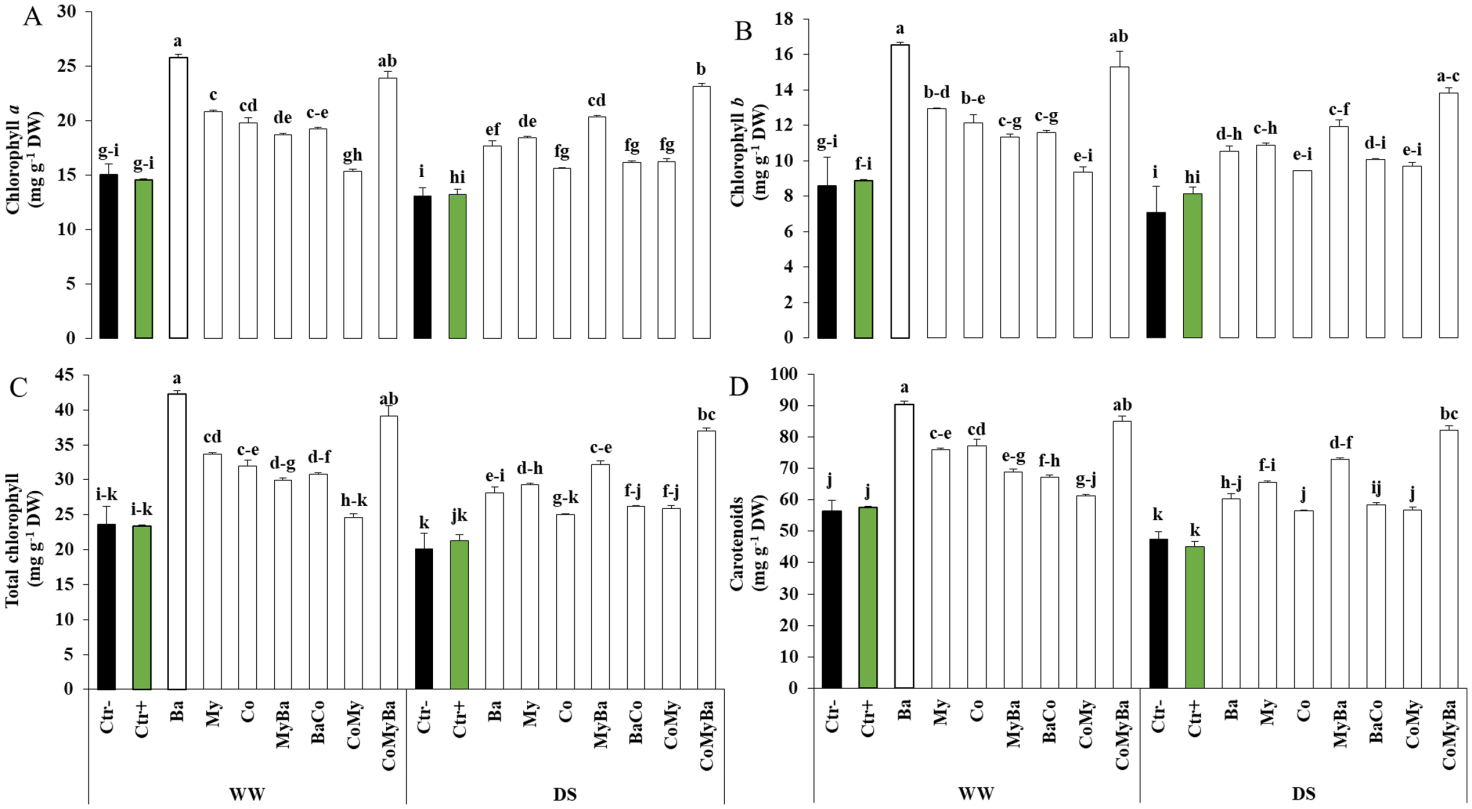
Effect of two irrigation levels and biostimulants on chlorophyll a **(A)**, chlorophyll b **(B)**, total chlorophyll **(C)** and carotenoids **(D)** of maize plants. WW, well-watered; DS, drought-stressed; Ctr−, untreated plants; Ctr+, plants treated by NPK; Ba, plant growth promoting rhizobacteria; My, arbuscular mycorrhizal fungi; Co, compost; MyBa, arbuscular mycorrhizal fungi and plant growth promoting rhizobacteria; BaCo, plant growth promoting rhizobacteria and compost; CoMy, compost and arbuscular mycorrhizal fungi; CoMyBa, compost and arbuscular mycorrhizal fungi and plant growth promoting rhizobacteria. Means (±SE; 5 biological replicates) followed by the same letters are not significantly different at *p* < 0.05 (Tukey’s HSD). Data were taken after harvesting.

It should also be noted that DS and the biostimulants modified the composition of photosynthetic pigments (chlorophyll [Chl] *a*, *b* and total Chl) and secondary metabolites (carotenoids) (Figure 5). DS caused a degradation of Chl *a* and *b* as well as total Chl. However, the single or combined application of biostimulants significantly improved (*p* < 0.05) these physiological parameters. In particular, the presence of Co in the soil in association with the two microbial consortia (CoMyBa) accumulated significantly (*p* < 0.05) more Chl *a*, Chl *b* and total Chl by 77, 95 and 83%, respectively compared with Ctr− under DS. In addition, under WW conditions, the leaves of plants treated with the bacterial consortium (Ba) without Co accumulated significantly 71% of Chl a, 92% of Chl b, 78% of total Chl and 60% of carotenoids compared to the unstressed and untreated Ctr− (Figures 5A–C). Similarly, biostimulants induced significant synthesis of carotenoids under DS, particularly in amended plots treated by My and Ba (73%) compared with MyBa (53%) without amendment versus Ctr− (Figure 5D).

#### 3.2.3 Leaf area (LA)

**Figure 6:**
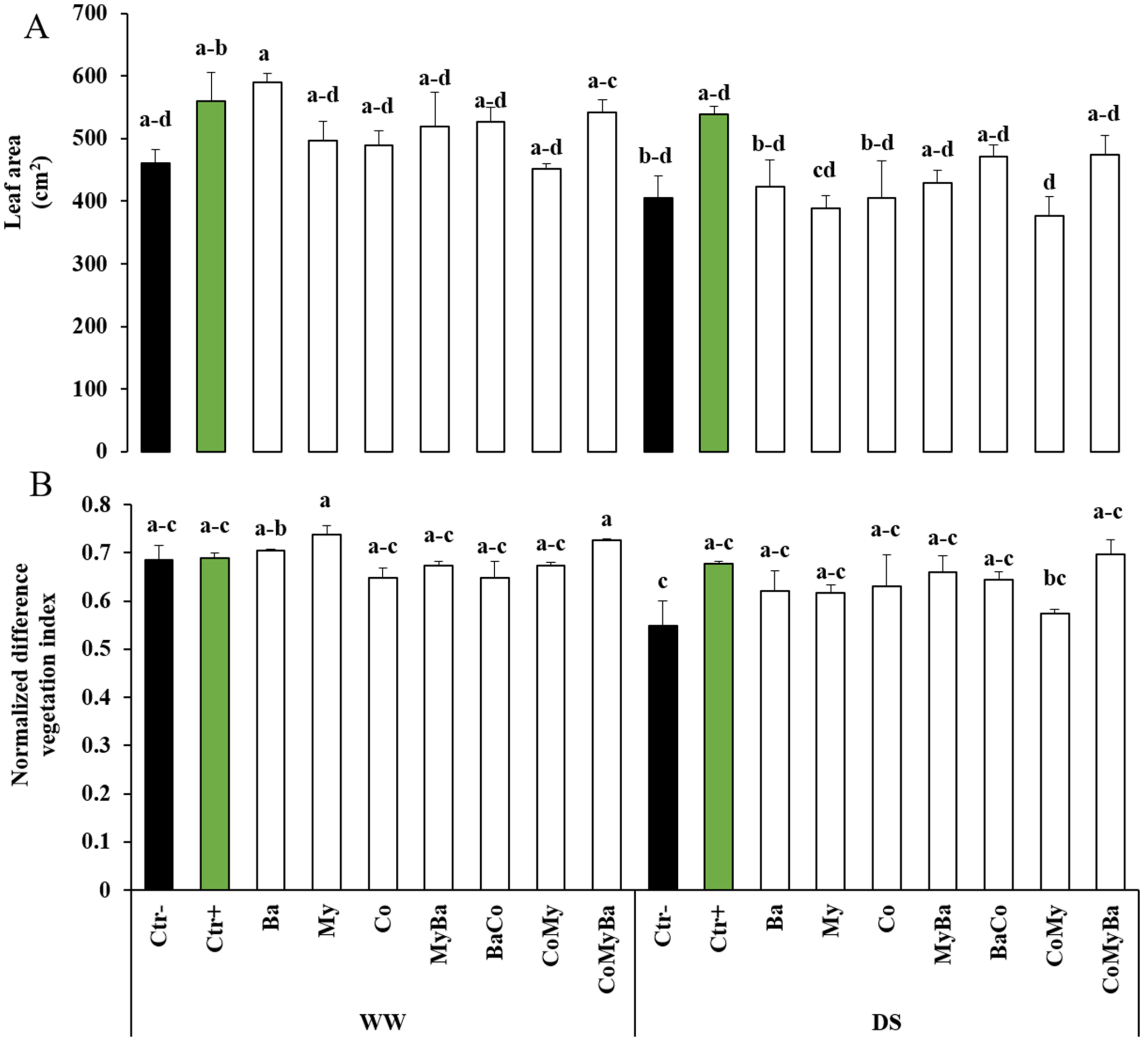
Effect of two irrigation levels and biostimulants on **(A)** leaf area and **(B)** normalized difference vegetation index of maize plants. WW, well-watered; DS, drought-stressed; Ctr−, untreated plants; Ctr+, plants treated by NPK; Ba, plant growth promoting rhizobacteria; My, arbuscular mycorrhizal fungi; Co, compost; MyBa, arbuscular mycorrhizal fungi and plant growth promoting rhizobacteria; BaCo, plant growth promoting rhizobacteria and compost; CoMy, compost and arbuscular mycorrhizal fungi; CoMyBa, compost and arbuscular mycorrhizal fungi and plant growth promoting rhizobacteria. Means (±SE; 5 biological replicates) followed by the same letters are not significantly different at *p* < 0.05 (Tukey’s HSD). Data were taken at the reproductive/milk stage.

A remarkable effect (*p* < 0.05) on LA was observed in maize plots treated with Ba alone (589.15 ± 15.34, 27%) or with Ctr+ (559.42 ± 45.84, 21.5%) or with CoMyBa (541.87 ± 20.19, 17%) under WW conditions. Under DS, the leaves of plants treated with the triple combination (CoMyBa) had a larger leaf area (474.07 ± 30.94, 16.8%) than those treated with microbes without Co and compared with Ctr− (Figure 6A).

#### 3.2.4 Normalized difference vegetation index (NDVI)

As NDVI is used in agriculture to assess the vigor and greenness of the vegetation, we tried to compare their variations with other physiological parameters for different treatments. The results in Figure 6B show that NDVI values fall where maize plants are irrigated at 50% ETc. On the other hand, under the same water regime, the incorporation of biostimulants into the soil improved NDVI values from 0.54 to 0.69. CoMyBa and Ctr+ were treatments that improved this index in maize plants by 27 and 23%, respectively, compared with all treatments without Co and Ctr−. On the contrary, under normal irrigation conditions, the highest NDVI values were observed in treated plants My, CoMyBa and Ba compared to Ctr− (Figure 6B).

### 3.3 Biochemical behavior

#### 3.3.1 Enzymatic activity

Reducing irrigation from 100% ETc to 50% ETc increased leaf enzyme activity. DS caused a significant increase in polyphenol oxidase (PPO) and catalase (CAT). High PPO enzyme activity was observed in Ba-treated plants than those treated by microorganisms in amended soil followed by Ctr− treated plants that were neither inoculated, fertilized nor amended under DS (Table 4). On the other hand, under the same conditions, CAT activity was highest in plants receiving CoMy, followed by untreated plants (Ctr−) and those inoculated with Ba (Table 4). Consequently, maize plants inoculated with My and Ba in amended soil had lower enzymatic activity of PPO and CAT.

**Table 4:** Effect of two irrigation levels and biostimulants on biochemical traits of maize plants.

Treatments	Proline (μmol g^−1^ DW)		Total soluble sugars (mg g^−1^ DW)		Polyphenoloxidase (nmol min^−1^ mg^−1^ proteins)		Catalase (UE min^−1^ mg^−1^ proteins)		Malonyldialdehyde (nmol g^−1^ DW)		Hydrogen peroxide (nmol g^−1^ DW)	
	WW	DS	WW	DS	WW	DS	WW	DS	WW	DS	WW	DS
Ctr−	20.3 ± 0.4 ^h^	33.2 ± 2.5 ^f-h^	673.2 ± 65.9 ^a-c^	465.1 ± 42.9 ^a-c^	0.169 ± 0.0 ^c^	0.859 ± 0.1 ^a^	56.0 ± 10.7 ^cd^	337.0 ± 146.6 ^ab^	46.7 ± 5.2 ^c^	96.2 ± 8.1 ^a-c^	0.015 ± 0.0 ^bc^	0.015 ± 9.8 ^bc^
Ctr+	37.9 ± 4.4 ^d-g^	64.0 ± 3.0 ^a^	617.9 ± 2.0 ^a-c^	745.1 ± 58.4 ^ab^	0.087 ± 0.0 ^c^	0.123 ± 0.0 ^c^	104.5 ± 29.7 ^b-d^	117.8 ± 47.8 ^b-d^	54.9 ± 1.3 ^bc^	68.1 ± 17.5 ^a-c^	0.012 ± 0.0 ^d-g^	0.007 ± 0.0 ^i^
Ba	37.0 ± 0.9 ^e-g^	54.5 ± 2.4 ^ab^	670.4 ± 59.6 ^a-c^	630.7 ± 85.0 ^a-c^	0.149 ± 0.0 ^a^	0.936 ± 0.0 ^c^	29.4 ± 6.47 ^d^	282.4 ± 47.7 ^a-c^	59.8 ± 16.9 ^a-c^	52.3 ± 1.0 ^c^	0.013 ± 0.0 ^c-e^	0.014 ± 4.6 ^b-d^
My	34.6 ± 6.8 ^e-h^	52.2 ± 1.9 ^a-c^	709.8 ± 99.6 ^a-c^	570.5 ± 28.0 ^a-c^	0.182 ± 0.0.0 ^c^	0.590 ± 0.2 ^ab^	144.1 ± 26.4 ^a-d^	165.8 ± 66.0 ^a-d^	109.6 ± 8.1 ^ab^	114.3 ± 3.4 ^a^	0.012 ± 0.0 ^f-h^	0.011 ± 6.6 ^gh^
Co	40.2 ± 0.7 ^c-f^	33.6 ± 0.9 ^e-h^	613.8 ± 30.1 ^a-c^	570.6 ± 84.3 ^a-c^	0.227 ± 0.0 ^bc^	0.197 ± 0.0 ^bc^	147.67 ± 43.6 ^a-d^	237.3 ± 11.6 ^a-d^	67.6 ± 17.2 ^a-c^	63.4 ± 10.1 ^a-c^	0.012 ± 0.0 ^e-h^	0.020 ± 0.0 ^a^
MyBa	40.9 ± 2.1 ^b-f^	37.2 ± 1.6 ^d-g^	546.4 ± 13.5 ^a-c^	679.1 ± 101.6 ^a-c^	0.113 ± 0.0 ^c^	0.103 ± 0.0 ^c^	76.4 ± 6.3 ^cd^	34.8 ± 11.7 ^cd^	78.4 ± 20.5 ^a-c^	68.6 ± 14.6 ^a-c^	0.011 ± 0.0 ^f-h^	0.015 ± 0.0 ^bc^
BaCo	25.4 ± 0.8 ^gh^	51.2 ± 0.2 ^a-d^	382.1 ± 28.8 ^c^	783.0 ± 43.6 ^a^	0.103 ± 0.0 ^c^	0.149 ± 0.0 ^c^	58.2 ± 23.1 ^cd^	78.5 ± 27.9 ^cd^	85.1 ± 4.1 ^a-c^	51.0 ± 2.6 ^c^	0.013 ± 5.7 ^d-f^	0.010 ± 0.0 ^h^
CoMy	32.8 ± 0.7 ^f-h^	47.5 ± 4.2 ^b-e^	552.1 ± 112.1 ^a-c^	470.6 ± 20.6 ^a-c^	0.263 ± 0.0 ^bc^	0.211 ± 0.0 ^bc^	140.5 ± 36.2 ^a-d^	379.9 ± 46.1 ^a^	92.1 ± 6.4 ^a-c^	51.3 ± 4.0 ^c^	0.012 ± 4.2 ^e-h^	0.016 ± 0.0 ^b^
CoMyBa	37.1 ± 1.6 ^d-g^	39.4 ± 2.1 ^c-g^	581.0 ± 47.2 ^a-c^	411.8 ± 84.8 ^bc^	0.374 ± 0.0 ^bc^	0.119 ± 0.0 ^c^	66.3 ± 21.9 ^cd^	37.8 ± 12.7 ^cd^	87.7 ± 3.8 ^a-c^	81.0 ± 6.5 ^a-c^	0.012 ± 2.9 ^e-h^	0.011 ± 2.6 ^f-h^

WW, well-watered; DS, drought-stressed; Ctr−, untreated plants; Ctr+, plants treated by NPK; Ba, plant growth promoting rhizobacteria; My, arbuscular mycorrhizal fungi; Co, compost; MyBa, arbuscular mycorrhizal fungi and plant growth promoting rhizobacteria; BaCo, plant growth promoting rhizobacteria and compost; CoMy, compost and arbuscular mycorrhizal fungi; CoMyBa, compost and arbuscular mycorrhizal fungi and plant growth promoting rhizobacteria.

Values are means ± SE for five biological replicates. The values of each column labeled with different letters indicate significant differences assessed by Tukey’s test (*p* < 0.05). Data were taken after harvesting.

#### 3.3.2 Osmotic adjustment regulation

From a metabolic point of view, DS and biostimulants have increased the content of osmotic substances. The greatest accumulation of proline (*p* < 0.05) was noted in plants treated with N-P-K (Ctr+) by 92.90% as well as those treated with biostimulants alone (Ba: 64.32% and My: 57.30%) or combined (BaCo: 54.27% and CoMy: 42.95%) under severe irrigation conditions (DS) compared with untreated plants (Table 4). Similarly, an effect of biostimulants on total soluble sugar (TSS) levels was noted mainly in plants grown in plots to which Co was added and inoculated with Ba (68.33%) followed by plants inoculated by MyBa (45.85%) compared with plants treated by double combination in amended soil and with Ctr− (Table 4).

#### 3.3.3 Stress markers

Stress markers such as hydrogen peroxide (H_2_O_2_) and malondialdehyde (MDA) were assessed to determine the damage induced by DS. Plants irrigated with 50% ETc accumulated high levels of H_2_O_2_, especially in plants treated with Co, Ctr− compared to the plants under 100 ETc. However, under the same water conditions, a significant reduction in H_2_O_2_ was observed in plants fertilized with N-P-K, BaCo and My, i.e., −51, −29, and −27%, respectively compared with plants treated by MyBa in amended soil and Ctr− (Table 4). Similar to H_2_O_2_, DS significantly increased MDA levels (*p* < 0.05). However, plants treated with BaCo, CoMy, Ba, Co, MyBa, and CoMyBa were able to attenuate MDA concentration by −46.91, −46.64, −45, −34, −28, and −15%, respectively compared to Ctr− (Table 4).

### 3.4 Soil properties and Glomalin content after harvest

Table 5 shows the results of the effects of the biostimulants applied on glomalin, available phosphorus (AP), nitrogen (N), total organic matter (TOM) and total organic carbon (TOC) under WW and DS conditions. Plots supplemented with Co alone or in association with microorganisms contained more TOM and TOC, in particular, CoMy (13.7%), BaCo (13.7%), Co (11.81%), and CoMyBa (7.87%) under WW than under Ctr−. On the other hand, under DS, the plots designated CoMy and CoMyBa had soils rich in TOM and TOC, 26 and 22% respectively, compared with the untreated plots. In addition, the biostimulants had a positive impact on the bioavailability of AP and N whatever the water regime imposed. For example, AP values reached their maximum in plots amended with Co (20%) and its combination with My (18%) under normal irrigation conditions. However, when irrigation was reduced to 50%, the percentage of AP fell to 65, 29, and 21% in the soils of plots treated with N-P-K (Ctr+), BaCo and CoMyBa, respectively, compared with Ctr−. An improvement in N was noted when biostimulants were introduced into the soil. Of all the treatments, CoMy, Ctr+, Co, and My appear to be the best in terms of N content, i.e., 100, 94, 38, and 38% compared with Ctr−. On the other hand, under DS conditions, the highest values were recorded in the plots inoculated with My compared with the Ctr− plots. Post-harvest soil pH varied between 7.1 and 8.4. Soil drying produced a slight increase in pH, especially in the plots amended and inoculated with the two microbial consortiums (CoMyBa) compared with all the treatments. Electrical conductivity (EC) was also modified by the effect of the biostimulants and the water regimes applied. Values for this parameter ranged from 0.4 to 1.15 mS/cm. Low EC values were recorded in plots inoculated with MyBa and My under DS and WW, respectively, compared with the other treatments. Glomalin levels changed from one treatment to another. Moreover, under normal irrigation conditions, glomalin was improved in plots treated with CoMy, Co and My i.e., 25.7, 25.4 and 20%, respectively compared with the untreated plots. However, under DS glomalin was increased only when the soil was fertilized with N-P-K compared with Ctr−. However, the incorporation of biostimulants, either separately or in combination, improved this physicochemical property, while a significant increase (*p* < 0.05) was observed when the soil was treated with CoMyBa under both favorable and unfavorable water regimes, with an improvement of 41 and 50%, respectively, compared with the soil from untreated plots. Compared with non-amended plots, the double microbial application in compost-treated plots improved cation exchange capacity (CEC) regardless of the water regime applied.

**Table 5:** Effect of two irrigation levels and biostimulants on soil physico-chemical properties.

Treatments	Glomalin (mg kg^−1^)		Available phosphorus (mg kg^−1^)		Nitrogen (%)		Total organic carbon (%)		Total organic matter (%)		Cation exchange capacity (meq/100 g)		Electrical conductivity (mS cm^−1^)		pH	
	WW	DS	WW	DS	WW	DS	WW	DS	WW	DS	WW	DS	WW	DS	WW	DS
Ctr−	2.1 ± 0.3 ^a^	2.6 ± 0.1 ^a^	38.3 ± 2.6 ^ab^	40.4 ± 2.6 ^ab^	0.06 ± 0.0 ^ab^	0.07 ± 0.0 ^ab^	2.0 ± 0.0 ^a-c^	1.7 ± 0.0 ^bc^	3.4 ± 0.0 ^a-c^	3.0 ± 0.1 ^bc^	4.4 ± 0.4 ^b^	4.4 ± 0.4 ^b^	1.1 ± 0.0 ^a^	0.4 ± 0.0 ^e-g^	7.8 ± 0.2 ^d-f^	7.8 ± 0.0 ^de^
Ctr+	2.2 ± 0.1 ^a^	2.7 ± 0.0 ^a^	40.8 ± 3.5 ^ab^	67.1 ± 11.8 ^a^	0.13 ± 0.0 ^a^	0.05 ± 0.0 ^ab^	2.0 ± 0.0 ^a-c^	1.8 ± 0.1 ^a-c^	3.4 ± 0.1 ^a-c^	3.2 ± 0.2 ^a-c^	5.9 ± 0.2 ^ab^	4.9 ± 0.2 ^ab^	0.6 ± 0.0 ^b^	0.5 ± 0.0 ^de^	7.1 ± 0.0 ^g^	8.0 ± 0.0 ^b-d^
Ba	2.0 ± 0.3 ^a^	2.4 ± 0.0 ^a^	39.8 ± 2.5 ^ab^	37.8 ± 3.3 ^b^	0.07 ± 0.0 ^ab^	0.01 ± 0.0 ^b^	1.9 ± 0.1 ^a-c^	1.9 ± 0.1 ^a-c^	3.3 ± 0.2 ^a-c^	3.3 ± 0.2 ^a-c^	4.9 ± 0.2 ^ab^	5.9 ± 0.4 ^ab^	0.4 ± 0.0 ^e-g^	0.4 ± 0.0 ^d-e^	7.7 ± 0.0 ^ef^	8.2 ± 0.0 ^a-c^
My	2.5 ± 0.1 ^a^	2.1 ± 0.2 ^a^	28.7 ± 1.3 ^b^	40.4 ± 8.4 ^ab^	0.09 ± 0.0 ^ab^	0.09 ± 0.0 ^ab^	1.9 ± 0.0 ^a-c^	1.9 ± 0.1 ^a-c^	3.2 ± 0.0 ^a-c^	3.2 ± 0.2 ^a-c^	6.2 ± 0.3 ^ab^	5.2 ± 0.4 ^ab^	0.4 ± 0.0 ^g^	0.4 ± 0.0 ^e-g^	7.7 ± 0.0 ^ef^	8.3 ± 0.0 ^ab^
Co	2.6 ± 0.0 ^a^	2.1 ± 0.0 ^a^	46.0 ± 0.6 ^ab^	44.6 ± 2.8 ^ab^	0.09 ± 0.0 ^ab^	0.04 ± 0.0 ^ab^	2.2 ± 0.0 ^ab^	2.0 ± 0.0 ^a-c^	3.9 ± 0.1 ^ab^	3.5 ± 0.1 ^a-c^	4.4 ± 0.4 ^b^	5.6 ± 0.2 ^ab^	0.5 ± 0.0 ^cd^	0.6 ± 0.0 ^b^	7.5 ± 0.0 ^f^	8.2 ± 0.0 ^a-c^
MyBa	2.0 ± 0.3 ^a^	1.7 ± 0.2 ^a^	27.1 ± 1.5 ^b^	39.7 ± 6.6 ^ab^	0.07 ± 0.0 ^ab^	0.02 ± 0.0 ^b^	1.8 ± 0.0 ^a-c^	1.6 ± 0.0 ^c^	3.2 ± 0.1 ^a-c^	2.8 ± 0.1 ^c^	4.9 ± 0.2 ^ab^	5.2 ± 0.4 ^ab^	0.4 ± 0.0 ^fg^	0.4 ± 0.0 ^g^	7.7 ± 0.0 ^d-f^	8.2 ± 0.0 ^ab^
BaCo	2.3 ± 0.0 ^a^	2.5 ± 0.0 ^a^	37.4 ± 7.0 ^b^	52.1 ± 5.1 ^ab^	0.06 ± 0.0 ^ab^	0.04 ± 0.0 ^ab^	2.3 ± 0.1 ^a^	2.0 ± 0.1 ^a-c^	3.9 ± 0.2 ^a^	3.5 ± 0.1 ^a-c^	5.6 ± 0.2 ^ab^	5.9 ± 0.2 ^ab^	0.6 ± 0.0 ^bc^	0.5 ± 0.0 ^de^	7.8 ± 0.0 ^ef^	8.2 ± 0.0 ^ab^
CoMy	2.6 ± 0.0 ^a^	2.2 ± 0.1 ^a^	45.4 ± 3.0 ^ab^	45.8 ± 8.8 ^ab^	0.13 ± 0.0 ^a^	0.05 ± 0.0 ^ab^	2.3 ± 0.0 ^a^	2.2 ± 0.1 ^ab^	3.9 ± 0.0 ^a^	3.9 ± 0.1 ^ab^	5.6 ± 0.2 ^ab^	5.9 ± 0.4 ^ab^	0.5 ± 0.0 ^de^	0.4 ± 0.0 ^d-g^	7.8 ± 0.0 ^d-f^	8.2 ± 0.0 ^a-c^
CoMyBa	2.1 ± 0.0 ^a^	2.3 ± 0.0 ^a^	33.2 ± 6.2 ^b^	49.3 ± 4.0 ^ab^	0.08 ± 0.0 ^ab^	0.05 ± 0.0 ^ab^	2.1 ± 0.0 ^ab^	2.1 ± 0.1 ^ab^	3.7 ± 0.0 ^a^	3.7 ± 0.1 ^ab^	6.3 ± 0.2 ^a^	6.7 ± 0.4 ^a^	0.4 ± 0.0 ^fg^	0.4 ± 0.0 ^fg^	7.9 ± 0.0 ^c-e^	8.4 ± 0.0 ^a^

WW, well-watered; DS, drought-stressed; Ctr−, untreated plants; Ctr+, plants treated by NPK; Ba, plant growth promoting rhizobacteria; My, arbuscular mycorrhizal fungi; Co, compost; MyBa, arbuscular mycorrhizal fungi and plant growth promoting rhizobacteria; BaCo, plant growth promoting rhizobacteria and compost; CoMy, compost and arbuscular mycorrhizal fungi; CoMyBa, compost and arbuscular mycorrhizal fungi and plant growth promoting rhizobacteria.

Values are means ± SE for five biological replicates. The values of each column labeled with different letters indicate significant differences assessed by Tukey’s test (*p* < 0.05). Data were taken after harvesting.

### 3.5 Pearson correlation and principal component analysis

**Figure 7:**
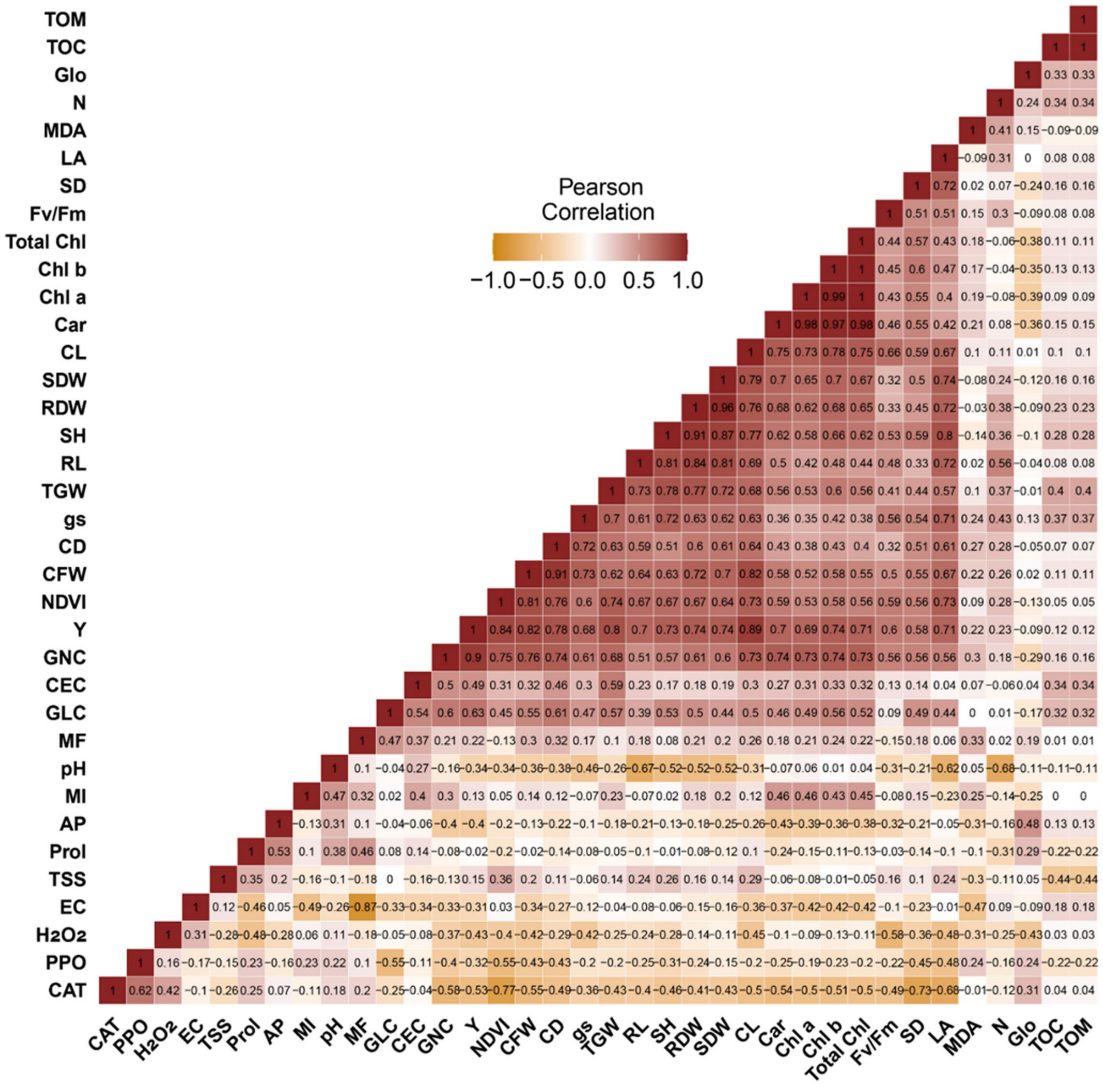
Pearson correlation analysis, describing the relationship between phenotypic, physiological and biochemical traits as well as soil physicochemical properties. SH, shoot height; RL, roots length; SDW, shoot dry weight; RDW, root dry weight; SD, stem diameter; CFW, cob fresh weight; CL, cob length; CD, cob diameter; GLC, grains lines number per cob; GNC, grains number per cob; TGW, thousand grains weight; Y, yield; Fv/Fm, chlorophyll fluorescence; gs, stomatal conductance; NDVI, normalized difference vegetation index; LA, leaf area; Chl *a*, chlorophyll *a*; Chl *b*, chlorophyll *b*; Total Chl, total chlorophyll; Car, carotenoids; MI, mycorrhizal intensity; MF, mycorrhizal frequency; H_2_O_2_, hydrogen peroxide; MDA, Malondialdehyde; PPO, polyphenoloxidase activity; CAT, catalase; TSS, total soluble sugars; Prol, proline; EC, electrical conductivity; TOM, total organic matter; TOC, total organic carbon; AP, available phosphorus; N, nitrogen; Glo, glomalin; CEC, cation exchange capacity.

The results in Figure 7 show that the parameters measured on the leaves and ears of maize, together with the physicochemical characteristics of the soil, are positively and negatively correlated, with Person coefficients ranging from 1 to −1. Indeed, a strong positive correlation (>0.5) was revealed between physiological parameters (g_s_, Fv/Fm, LA, NDVI, Chl a, Chl b, Total Chl, and Car) and phenotypic traits linked to biomass and maize grain yield (Y, TGW, GLC, GNC, CFW, CD, CL, RDW and SDW). MI also correlates positively with physiological parameters. Soil CEC also aligns positively with TGW with a coefficient of 0.59. Conversely, a negative correlation was observed between H_2_O_2_ and the phenotypic and physiological traits of maize plants.

**Figure 8:**
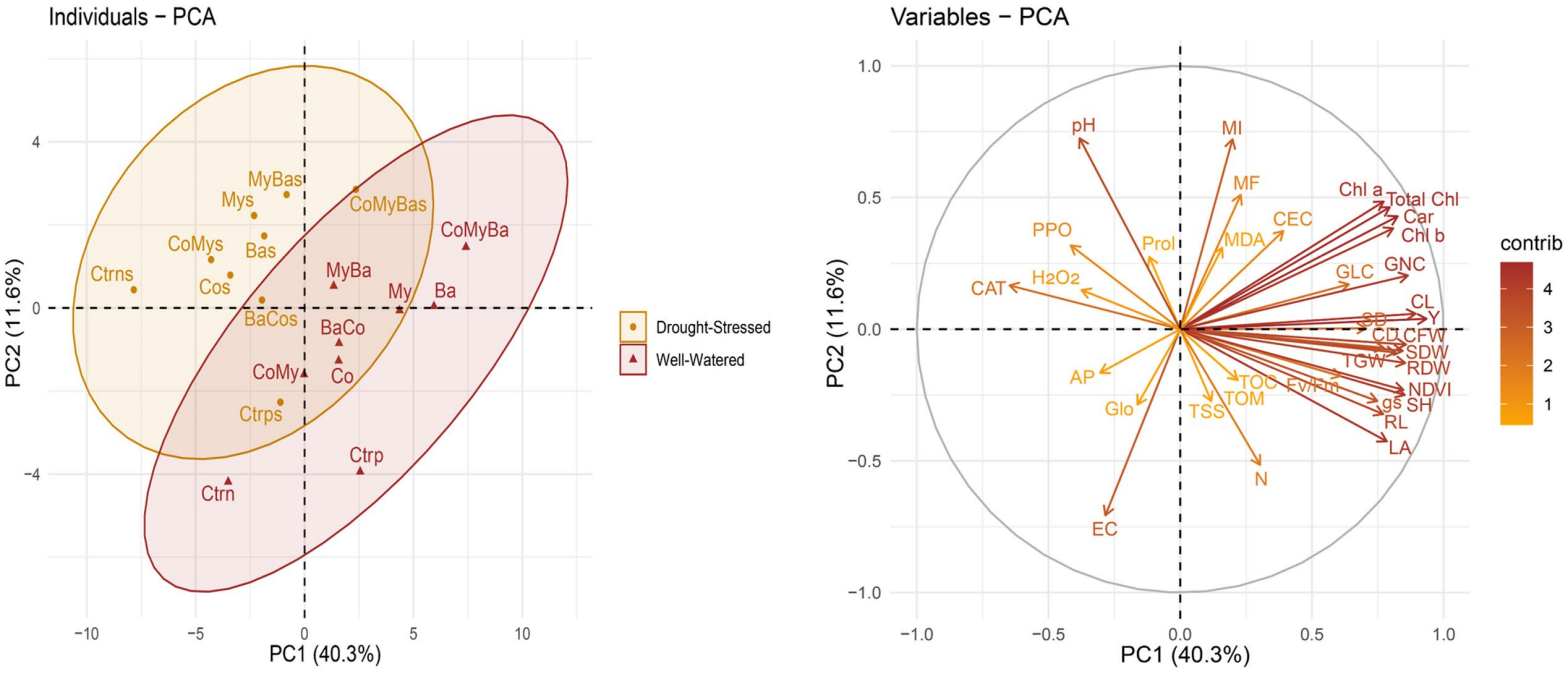
Principal component analysis (PCA: individuals and variables) of all the parameters measured for maize under two irrigation levels and biostimulants. Ctrn, untreated plants; Ctrp, plants treated by NPK; Ba, plant growth promoting rhizobacteria; My, arbuscular mycorrhizal fungi; Co, compost; MyBa, arbuscular mycorrhizal fungi and plant growth promoting rhizobacteria; BaCo, plant growth promoting rhizobacteria and compost; CoMy, compost and arbuscular mycorrhizal fungi; CoMyBa, compost and arbuscular mycorrhizal fungi and plant growth promoting rhizobacteria. SH, shoot height; RL, roots length; SDW, shoot dry weight; RDW, root dry weight; SD, stem diameter; CFW, cob fresh weight; CL, cob length; CD, cob diameter; GLC, grains lines number per cob; GNC, grains number per cob; TGW, thousand grains weight; Y, yield; Fv/Fm, chlorophyll fluorescence; gs, stomatal conductance; NDVI, normalized difference vegetation index; LA, leaf area; Chl *a*, chlorophyll *a*; Chl *b*, chlorophyll *b*; Total Chl, total chlorophyll; Car, carotenoids; MI, mycorrhizal intensity; MF, mycorrhizal frequency; H_2_O_2_, hydrogen peroxide; MDA, Malondialdehyde; PPO, polyphenoloxidase activity; CAT, catalase; TSS, total soluble sugars; Prol, proline; EC, electrical conductivity; TOM, total organic matter; TOC, total organic carbon; AP, available phosphorus; N, nitrogen; Glo, glomalin; CEC, cation exchange capacity; s, stress.

Principal component analysis (PCA) revealed a total variability of 51.9%, with PC1 = 40.3% and PC2 = 11.6% for all the parameters evaluated in maize plants and soil treated or not with biostimulants under normal or stressed conditions (Figure 8). Under these conditions, biostimulants applied as a single or combined action had a positive effect on physiological, biochemical, growth and yield parameters. PCA of individuals detected along the PC1 axis, a positive association between the Ba, My, MyBa, and CoMyBa treatments under normal irrigation conditions and the CoMyBas treatment under water stress conditions. This aligns with PCA variables on the same axis through a strong contribution (>3) observed concerning the effect on growth parameters (Y, TGW, GLC, GNC, CFW, CD, CL, RDW, and SDW), physiology (gs, Fv/Fm, LA, NDVI, Chl a, Chl b, Total Chl, and Car) as well as mycorrhization intensity.

### 3.6 Hierarchical ascending classification and parallel coordinates plots

**Figure 9:**
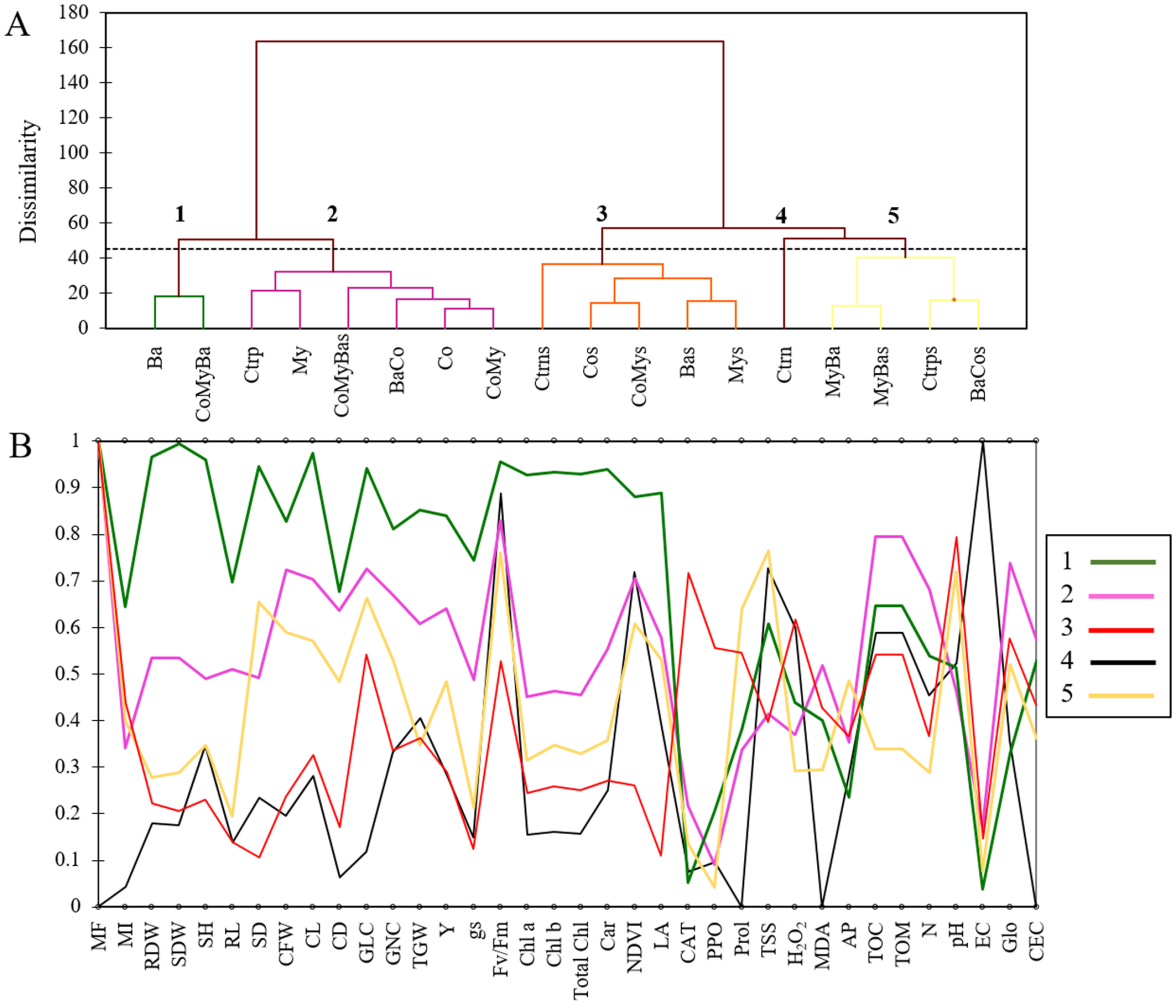
**(A)** Hierarchical ascending classification and **(B)** parallel coordinate plot analyses of all the parameters measured for maize under two irrigation levels and biostimulants. Ctrn, untreated plants; Ctrp, plants treated by NPK; Ba, plant growth promoting rhizobacteria; My, arbuscular mycorrhizal fungi; Co, compost; MyBa, arbuscular mycorrhizal fungi and plant growth promoting rhizobacteria; BaCo, plant growth promoting rhizobacteria and compost; CoMy, compost and arbuscular mycorrhizal fungi; CoMyBa, compost and arbuscular mycorrhizal fungi and plant growth promoting rhizobacteria. SH, shoot height; RL, roots length; SDW, shoot dry weight; RDW, root dry weight; SD, stem diameter; CFW, cob fresh weight; CL, cob length; CD, cob diameter; GLC, grains lines number per cob; GNC, grains number per cob; TGW, thousand grains weight; Y, yield; Fv/Fm, chlorophyll fluorescence; gs, stomatal conductance; NDVI, normalized difference vegetation index; LA, leaf area; Chl *a*, chlorophyll *a*; Chl *b*, chlorophyll *b*; Total Chl, total chlorophyll; Car, carotenoids; MI, mycorrhizal intensity; MF, mycorrhizal frequency; H_2_O_2_, hydrogen peroxide; MDA, Malondialdehyde; PPO, polyphenoloxidase activity; CAT, catalase; TSS, total soluble sugars; Prol, proline; EC, electrical conductivity; TOM, total organic matter; TOC, total organic carbon; AP, available phosphorus; N, nitrogen; Glo, glomalin; CEC, cation exchange capacity; s, stress.

Hierarchical ascending classification (HAC) was performed on the average of the various parameters studied per treatment in order to group them into homogeneous classes. Analysis of this dendrogram shows that the 18 treatments were divided into five (5) groups. 1: Ba and CoMyBa; 2: Ctrp, My, CoMyBas, BaCo, Co and CoMy; 3: Ctrns, Cos, CoMys, Bas and Mys; 4: Ctrn; 5: MyBa, MyBas, Ctrps and BaCos (Figure 9A).

Analysis of parallel coordinate plot (PCP) in relation with HAC showed the origin of dissimilarity between treatments applied under normal or stressed irrigation conditions based on the parameters studied. Groups 1 (green) and 2 (violet) represent treatments that increased growth and physiological parameters under normal irrigation conditions, compared with group 4 (Ctrn). It should be noted that the CoMyBas treatment was able to improve these parameters under drought stress conditions. Group 5, on the other hand, was responsible for an improvement in yield-related parameters as well as osmolyte accumulation (Prol and TSS) under water stress conditions compared with group 3, which included Ctrns (Figure 9B).

## 4 Discussion

The resilient management of agrosystems in arid and semi-arid zones prone to drought could be linked to the exploitation of local resources, such as the implementation of protocols based on the dual application of beneficial microbial inocula to compost-amended soil. The present study describes the phenotypic, physiological and biochemical responses of maize plants inoculated with beneficial microorganisms and grown in the presence and absence of compost under conditions of 50 and 100% ETc. Our findings revealed that the inoculation and amendment of the cultivated plots with biostimulants significantly improved phenotypic traits under normal and stressed conditions. This effect was remarkable in maize plants treated with bacterial consortium applied alone or in combination with mycorrhizal consortium in compost-amended plots. These results are in line with several studies (Baslam et al., 2011; Ben-Laouane et al., 2021; Inbaraj, 2021; Meddich, 2022). The current advantage may result from the dissolution of phosphate and potassium, the complexation of iron and the consequent increased bioavailability of mineral elements, which improves plant growth under adverse conditions (Armada et al., 2015; Mahdi et al., 2021; Slimani et al., 2022). Improved growth and biomass accumulation in compost-amended inoculated maize plants could be explained by the ability of plant growth-promoting rhizobacteria (PGPR) to act through their direct and indirect mechanisms on plants, in particular the property of phosphate solubilization (Masciarelli et al., 2014; Chakraborty et al., 2018). The obtained results reveal that the presence of PGPR in the soil stimulates root and aerial elongation of maize plants regardless of the moisture in the soil. This could be due to the ability of PGPR to activate the biosynthetic pathways of growth regulators, in particular, auxin where tryptophan (precursor) is converted to indole-3-pyruvic acid by a tryptophan aminotransferase (Noor et al., 2023). Auxin stimulates root elongation and root surface area, leading to increased chances of maximum uptake of mineral elements and water under drought conditions (El-sharabasy and Ragab, 2009; Hayat et al., 2010; Vacheron et al., 2013). As long as our bacterial consortium is interacting with several other microorganisms in the experimental site, there may be a control of pathogens that can damage the maize crop via the release of antimicrobial and/or antifungal substances (Meena et al., 2020). Another hypothesis is that 1-aminocyclopropane-1-carboxylic acid (ACC), the direct precursor of ethylene in plants, is hydrolyzed, reducing ethylene levels, including tolerance to drought stress (Habben et al., 2014). In addition, these beneficial contributions may be conditioned by inoculation with the two bacteria (*Bacillus* sp. and *Bacillus subtilis*) in consortium bacteria. It has been shown that consortium application of the microorganisms further improved phenotypic, physiological biochemical and nutritional traits (Saleem et al., 2021). In addition to the PGPR, compost with its composition helps plants maintain their physiological and water status under drought conditions (Mbarki et al., 2018). Compost enriches the soil with mineral elements (P, N, K, etc.), bioactive substances and humic organic matter (Steiner et al., 2007; Yu et al., 2019). This provides a suitable matrix for root expansion and invasion of large soil volumes, resulting in high absorption of water and mineral elements, which improves plant growth and development (Xu et al., 2018). Thanks to its structure, compost increases water retention in drought-affected soils (Mohd Dolit et al., 2022). Indirectly, organic amendments stimulate plant growth by encouraging the proliferation of plant-beneficial microorganisms in the soil (Viti et al., 2010; Liu X. et al., 2022). Compost mitigates the oxidative damage caused by drought stress by strengthening the antioxidant system, increasing photosynthetic activity and improving stomatal conductance (Anli et al., 2020). It increases plant tolerance to drought stress through several mechanisms including (1) improved water retention in the soil, (2) increased nutrient retention capacity, (3) improved soil structure, (4) stimulation of the growth of microorganisms beneficial to plant growth, (5) availability of bioactive compounds such as humic substances capable of triggering antioxidant enzyme signaling pathways, and (6) significant supply of nutrients essential to the proper functioning of the photosynthetic apparatus (Duong et al., 2012; Sharma et al., 2017; Liu et al., 2018; Adamipour et al., 2020; Meddich, 2022; Mohd Dolit et al., 2022; Omara et al., 2022). It has been suggested that drought negatively reduces maize yield (Shirinbayan et al., 2019). This is because drought stress generally reduces nutrient uptake by roots and transfer to shoots, as well as transpiration rates are limited (Bárzana and Carvajal, 2020). During the maize crop cycle of our experiment, the yields obtained with normal (100% ETc) and incomplete (50% ETc) irrigation are equivalent to 480 and 240 mm of water consumed, respectively. Various geographical and climatic factors, such as temperature, atmospheric humidity, precipitation, sunshine and altitude, influence the amount of water a plant needs for optimal development (Masia et al., 2021). In our case, maize yield-related parameters such as fresh ear weight, diameter, elongation, thousand kernel weight and ton yield per hectare were negatively affected by drought stress. Moreover, in this study, the yields ranged from 3.86 Mg h^−1^ for untreated plots (i.e., unfertilized, uninoculated and unamended) to 10.56 Mg h^−1^ for plots with compost, PGPR and AMF added to the soil under drought stress conditions. AMF have been reported to enhance plant growth under conditions of abiotic stress (Mitra et al., 2021; Akensous et al., 2022; Ouhaddou et al., 2023b; Silva et al., 2023). Our investigations show that drought stress encouraged root colonization by mycorrhizal structures, notably hyphae and vesicles. This colonization was more aggressive in particular when the soil was amended with compost and inoculated with the bacterial consortium under limited water conditions. Such an increase is in line with the increase in agronomic, physiological and biochemical parameters in particular under 50% ETc. Compost can enhance mycorrhizal structures by improving the rhizosphere environment, supporting the persistence of mycorrhizal symbiosis, and ultimately benefiting plant health and resilience to drought stress. Enhancing root mycorrhization could be due to the presence of humic substances in compost (Pinos et al., 2019). In addition, such a tripartite combination performed better in improving chlorophyll a and b content, leaf area and normalized difference vegetation index (NDVI). These findings are in agreement with the results of Boutasknit et al. (2021) on the effect of single or combined application of biostimulants on carob plants. Similarly, catalase (CAT) enzymatic activity reached its maximum in maize plants mycorrhized in compost-amended soil. It has been shown that AMF to sesame plant roots induced high antioxidant enzyme activity and increased osmolyte accumulation, thereby increasing tolerance to drought and improving growth (Begum et al., 2020; Gholinezhad et al., 2020). In our study, yield and thousand kernel weight were positively affected when maize plants were inoculated with AMF and/or PGPR. This could be due to good assimilation of mineral elements and water via AMF in favor of their extended hyphae under drought stress conditions (Zou et al., 2014; Zhang et al., 2018). From a genetic point of view, overexpression of genes encoding aquaporins was revealed on the surface of Glomus hyphae suggesting a better water supply to the plant (Li et al., 2013). Several factors can control mycorrhizal symbiosis: soil texture, plant genotype, environmental conditions and biotic factors (Berger and Gutjahr, 2021).

The reduced efficiency of the photosynthetic apparatus under drought stress is closely linked to the imbalance between light capture and utilization, the destruction of the chloroplast structure, low activity of the enzyme responsible for CO_2_ fixation (Rubisco), and increased chlorophyllase activity (Sallam et al., 2019). In interaction with drought stress, biostimulants can improve physiological traits such as chlorophyll fluorescence, stomatal conductance, water potential, leaf area, and photosynthetic pigment synthesis (Qiao et al., 2011; Abbaspour et al., 2012; Liu et al., 2015; Malik et al., 2020; Soussani et al., 2023). Improvement of stomatal conductance under drought stress by biostimulants applied alone or in combination can result in adequate CO_2_ uptake, and consequently protein and sugar synthesis (Nie et al., 2015). In this study, it was demonstrated that double-inoculated maize plants grown in compost-amended soil showed a significant increase in chlorophyll a and b content as well as total chlorophyll under stress due to the 50% reduction of crop water requirement. This could be explained by high photosynthetic enzyme activity (Baslam et al., 2020). The improvement in chlorophyll fluorescence is reflected in the proper functioning of photosynthetic pigments in the reaction center, especially at photosystem II (Ye et al., 2019). Numerous studies have confirmed the positive effect of biostimulants on the leaf area of various plants such as maize, wheat, lettuce, tomato and barley (Ali et al., 2011; Aini et al., 2019; Hafez et al., 2020; Kakabouki et al., 2020; Othmani et al., 2020). Treatment of plants with biostimulants under conditions of water stress can protect photosynthetic structures by neutralizing reactive oxygen species through increased antioxidant enzyme activity (Mo et al., 2016). Similarly, our study showed that inoculation of maize plants with PGPR separately or combined with AMF, particularly in compost-amended soil increased the leaf area of maize plants under 50% evapotranspiration. Researchers have explained this increase by cell expansion under the effect of certain phytohormones, notably cytokinins (Franklin, 2009; Zhao et al., 2021). It has been demonstrated that larger leaf areas intercept more sunlight, providing more energy for photosynthesis (Patil et al., 2018). Greater leaf area and light energy allow for more photosynthetic activity, leading to increased production of carbohydrates and plant growth (Wang et al., 2015; Patil et al., 2018). One of the strategies used by plants to escape drought stress is osmotic adjustment, generated by a significant accumulation of osmolytic substances, notably proline, glycine betaine, organic acids and total soluble sugars (Quilambo, 2004; Chen and Jiang, 2010; Choudhary et al., 2022). These substances help the plant maintain its water balance by creating a potential difference on either side of the cell membrane, which calls up water from the soil to the roots (Iqbal and Nazar, 2015; Ghosh et al., 2021). During periods of water stress, osmolytes such as sugars can act as signals to regulate stomatal closure, thereby reducing water loss through transpiration, while still maintaining an adequate supply of carbon dioxide for photosynthesis (Wang et al., 2022). Proline plays an important role in determining protein and membrane structure, as well as the scavenging of reactive oxygen species (Ashraf and Foolad, 2007). In our work, drought stress induced proline accumulation especially under biostimulant application as in plants grown in plots inoculated with AMF or PGPR. This accumulation is often due to overexpression of the gene encoding 1-pyrroline-5-carboxylate synthetase (P5CS) under abiotic stress conditions (Chun et al., 2018). Under drought stress, the high concentration of proline in AMF-inoculated plants leads to increased leaf water content, enhanced photosynthetic activity and significant modulation of sugars (Foyer et al., 2017). In this context, our study also revealed a significant accumulation of total leaf-soluble sugars in plants inoculated with bacteria and amended with compost. Indeed, studies have confirmed the role of sugars in the protection and osmotic regulation of plants in dry soils (Nayer and Reza, 2008). Nemati et al. (2018) found that drought-tolerant seedlings increase the synthesis of sugars, in particular, sucrose at the metabolic and gene expression level to increase energy savings and regulate cellular metabolism. Plant protection against drought is not only limited to the accumulation of osmolytes but also by an enzymatic antioxidant system armed with several enzymes, as our work showed. The synthesis of secondary metabolites is also one of the strategies used by plants to escape drought (Yadav et al., 2021). The carotenoid pathway is an example of a metabolite that becomes highly active when the plant is in a drought situation. As our work showed, biostimulants are able to increase foliar carotenoids in maize plants under drought stress. Plots of maize plants treated with the tripartite combination (CoMyBa) were the most distinctive for improved carotenoid content compared with plants whose plots were not treated. Similarly, Begum et al. (2020) showed that inoculation of maize plants with *Glomus versiforme* improved carotenoid composition by 89.80% under drought stress conditions compared with non-mycorrhized plants. At the genetic level, Zhang et al. (2021), confirmed that carotenoid synthesis in carrots was linked to gene 9-cis-epoxycarotenoid dioxygenase expression under severe water limitation. By acting on the physiological processes of plants, biostimulants induce positive changes in the soil, creating an environment suitable for crop growth and development. Drought stress in arid and semi-arid regions, as reported by several researchers, negatively affects a range of soil characteristics including available phosphorus (AP), nitrogen (N), total organic matter and carbon (TOM and TOC), and cation exchange capacity (CEC). Tolerance to drought stress may begin at the soil level before reaching the above-ground parts, via the positive effect of biostimulants on soil physico-chemical properties. For this reason, our study also focused on soil measurements. Our results showed that soil enrichment with compost and/or AMF and/or PGPR increased total organic matter, AP, CEC and glomalin. Organic amendments increased the bioavailability of mineral elements, notably AP, N, potassium (K) and magnesium (Mg), which are beneficial to the growth and tolerance of various crops to abiotic stresses (Benaffari et al., 2022). The presence of compost and/or microorganisms in the soil had a huge impact on the formation and stabilization of soil agglomerates, improved soil CEC and increased nutrient retention, thereby increasing the uptake of nitrogen, phosphorus and potassium by cereals under drought stress (Seneviratne et al., 2011; Sharma et al., 2017; Liu X. Q. et al., 2022; Ikan et al., 2023). The high CEC values in our study site in particular, plots receiving all three biostimulants at the same time, translates into good soil fertility, and good retention of mineral elements. This also means that the clay-humus complex retains cations such as calcium (Ca^2+^), sodium (Na^+^) and potassium (K^+^) and releases them into the soil solution according to its pH (Seneviratne et al., 2011). It has been postulated by several scientific reports that amending soils with different types of biochar improves their CEC, and therefore their fertilities, which improves plant growth (Agegnehu et al., 2016). Artiola et al. (2012) suggested that the increased water retention capacity of soil under drought stress could be due to the higher CEC and porous structure of biochar. The high levels of AP observed in the soil after harvesting maize plants, especially in plots treated with compost in association or not with microbes, may be at the origin of the compost itself in a direct way or through the action of PGPRs in an indirect way. The stability of soil aggregates plays an important role in soil quality, protecting soil organic matter from microbial decomposition and preventing the degradation of soil structure. The stability of the soil’s aggregates is crucial to its quality because it shields the organic matter from microbial degradation and prevents the structure of the soil from deteriorating. The improvement of soil quality is correlated with some microbe secretions, such as glomalin released by AMF (Wright et al., 1998). This substance’s hydrophobic characteristic enables it to stabilize soil particle formation (Singh, 2012). It has been shown that there is a strong correlation between the content of glomalin and the stability of soil aggregates (Wright and Anderson, 2000). Our findings show that the glomalin improved in the presence of compost and AMF under 100% ETc. In this context, Zhang et al. (2014), revealed that the stability of soil aggregates was enhanced by the addition of organic amendment thanks to soil particle binding agents, in particular glomalin (Zhang et al., 2014). It has been reported that glomalin plays a vital part in the soil that allows plants to tolerate abiotic stress (Chi et al., 2018). This may be due to the formation of a hydrophobic layer on the surface of the aggregates, preventing the loss of water and nutrients from plants exposed to abiotic stress (Wu et al., 2008). Consequently, it is important to point out that the biostimulants applied in this study under drought stress, respond positively in two facets, in the soil and in the maize plants.

Factors influencing the adoption and success of biostimulants applications include local agricultural practices, which vary widely and impact the integration of new technologies like biostimulants (Shahrajabian et al., 2023). Land management practices, such as soil preparation and fertility management, also play a crucial role in determining the effectiveness of biostimulants (Castiglione et al., 2021). Socio-economic conditions, including economic viability, farmer attitudes, and market dynamics, significantly influence adoption rates (Wazeer et al., 2024). Understanding and addressing these diverse factors are essential for effectively implementing biostimulants strategies tailored to local agricultural contexts.

## 5 Conclusion

In light of the results obtained, it is crucial to point out that the introduction of biostimulants into agrosystems in arid and semi-arid regions, using the technology of combining compost, Arbuscular mycorrhizal fungi (AMF) and plant growth-promoting rhizobacteria (PGPR), proves to be an ecological approach aimed at protecting maize plants against drought damage. The effect of biostimulants on maize plants under two levels of water based on crop evapotranspiration (ETc), 100 and 50%, was evaluated by assessing phenotypic, physiological, biochemical and nutritional traits. Inoculation with microbes and/or organic amendment boosted the growth, thousand-seed weight and yield of maize plants under drought stress conditions by positively affecting stomatal conductance, photosynthetic pigment content, leaf area and the normalized difference vegetation index (NDVI). In other words, tolerance to drought stress via the application of biostimulants is at the origin of the attenuation of stress markers accompanied by an accumulation of osmolytes and the reinforcement of antioxidant enzyme activity. Hierarchical ascending classification and parallel coordinate plot analyses results showed that PGPR alone and PGPR + Compost + AMF are the best-performing treatments in terms of tolerance to drought stress, compared with all other treatments. In addition, biostimulants positively modified soil quality, which in turn improved maize plant growth under drought stress. Despite these positive statements, certain limitations need to be taken into account. The results are specific to these geographical conditions, and their generalization to other regions or climates requires further study. In addition, the study was carried out over a relatively short period, which limits the assessment of long-term effects on soil health and the sustainability of practices. The efficacy of biostimulants on crops other than maize also remains to be explored. Furthermore, the complex interactions between PGPRs, AMFs and compost may vary according to environmental conditions, requiring further research on a large scale to optimize these synergies. Finally, certain aspects, such as the impact on soil biodiversity or long-term nutrient cycles, have not been studied and merit further investigation. We, therefore, advise farmers to turn to this kind of sustainable biological technology to preserve agricultural ecosystems against the degradation caused by drought waves.

## Data Availability

The original contributions presented in the study are included in the article/Supplementary material, further inquiries can be directed to the corresponding authors.

## Footnotes

^1^https://invam.ku.edu/species
