# Supplementary material for: Investigation of the impact of dual inoculations of arbuscular mycorrhizal fungi and plant growth-promoting rhizobacteria on drought tolerance of maize grown in a compost-amended field under Mediterranean conditions
Source: Front Microbiol. 2024 Oct 9;15:1432637. doi: 10.3389/fmicb.2024.1432637 (PMC11519983; doi:10.3389/fmicb.2024.1432637)
Supplement: Supplementary file 1 [file Table_1.pdf]

Supplementary Table 1: Arbuscular mycorrhizal fungi species used

Arbuscular mycorrhizal fungi species
Acaulospora delicata, *Acaulospora leavis*, *Acaulospora* sp.<br>*Claroideoglomus claroideum.*<br>*Glomus aggregatum*, *G. claroides*, *G.clarum*, *G. deserticola*, *G. heterosporum*, *G. macrocarpum*, *G. microcarpum*, *Glomus* sp, *G. versiforme.*<br>*Rhizophagus intraradices.*<br>*Pacispora boliviana.*
